# SRSF1 Is Required for Mitochondrial Homeostasis and Thermogenic Function in Brown Adipocytes Through its Control of Ndufs3 Splicing

**DOI:** 10.1002/advs.202306871

**Published:** 2024-04-03

**Authors:** Ningyang Yuan, Lei Shen, Qian Peng, Rula Sha, Zhenzhen Wang, Zhiqi Xie, Xue You, Ying Feng

**Affiliations:** ^1^ CAS Key Laboratory of Nutrition, Metabolism and Food Safety Shanghai Institute of Nutrition and Health University of Chinese Academy of Sciences Chinese Academy of Sciences Shanghai 200031 China; ^2^ Lin He's Academician Workstation of New Medicine and Clinical Translation in Jining Medical University Jining Medical University Jining 272067 China; ^3^ Department of General Surgery Zhongshan Hospital Fudan University Shanghai 200032 China

**Keywords:** dysregulatred splicing, impaired thermogenesis, mitochondria dysfunction, splicing regulator, whitening of brown adipocyte tissue

## Abstract

RNA splicing dysregulation and the involvement of specific splicing factors are emerging as common factors in both obesity and metabolic disorders. The study provides compelling evidence that the absence of the splicing factor SRSF1 in mature adipocytes results in whitening of brown adipocyte tissue (BAT) and impaired thermogenesis, along with the inhibition of white adipose tissue browning in mice. Combining single‐nucleus RNA sequencing with transmission electron microscopy, it is observed that the transformation of BAT cell types is associated with dysfunctional mitochondria, and SRSF1 deficiency leads to degenerated and fragmented mitochondria within BAT. The results demonstrate that SRSF1 effectively binds to constitutive exon 6 of Ndufs3 pre‐mRNA and promotes its inclusion. Conversely, the deficiency of SRSF1 results in impaired splicing of Ndufs3, leading to reduced levels of functional proteins that are essential for mitochondrial complex I assembly and activity. Consequently, this deficiency disrupts mitochondrial integrity, ultimately compromising the thermogenic capacity of BAT. These findings illuminate a novel role for SRSF1 in influencing mitochondrial function and BAT thermogenesis through its regulation of Ndufs3 splicing within BAT.

## Graphical abstract

Eliminating SRSF1 genetically triggers the whitening of brown adipocyte tissue (BAT) and compromises its thermogenic function, consequently causing metabolic disorders in mice. Combining single‐nucleus RNA sequencing with transmission electron microscopy, the study highlights that the absence of SRSF1 disrupts mitochondrial homeostasis, leading to an overproduction of mitochondrial reactive oxygen species in BAT due to alterations in the splicing of Ndufs3.

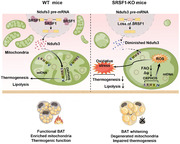

## 1 Introduction

Obesity, characterized by the accumulation of excess body fat resulting from an imbalance between energy intake and energy expenditure (EE),^[^ ^1^ ^]^ underscores the significance of enhancing EE in key metabolic organs like adipose tissue as a potential strategy for obesity prevention.^[^ ^2^ ^]^ Adipose tissue comprises two primary types: white adipocyte tissue (WAT), functioning primarily as an energy reservoir, and BAT, responsible for non‐shivering thermogenesis and capable of utilizing surplus lipids and glucose for energy expenditure.^[^ ^3^ ^]^ Studies have demonstrated that external stimuli can induce the transformation of a subset of WAT cells into brown‐like adipocytes, referred to as beige cells, in a process known as white fat “browning”.^[^ ^2^ ^]^ Notably, substantial quantities of metabolically active BAT have been observed in adult humans, underscoring the potential of stimulating the development and function of both brown and beige fat as a promising approach for combatting obesity.^[^ ^4^ ^]^

Proper mitochondrial function is essential for the metabolic stability and performance of BAT.^[^ ^5^ ^]^ The mitochondrial respiratory chain, encompassing the mitochondrial electron transport chain (mETC) and Fo‐F1ATP synthase, constitutes a series of protein complexes (complexes I, II, III, IV, and V) situated within the within the inner mitochondrial membrane (IMM), playing a central role in oxidative phosphorylation (OXPHOS). Electrons required for OXPHOS are sourced from metabolic pathways involving carbohydrates, lipids, and amino acids, and are channeled into the mETC.^[^ ^6^ ^]^ Complex I (CI), composed of 45 subunits encoded by both mitochondrial DNA (mtDNA) and nuclear DNA (nDNA), serves as the primary and intricate enzyme within the respiratory chain. Its function involves catalyzing the transfer of electrons from NADH oxidation to ubiquinone.^[^ ^1^, ^6^, ^7^ ^]^ Additionally, CI plays a crucial role in generating reactive oxygen species (ROS). ROS are normal byproducts of respiration and are necessary for intracellular signaling pathways.^[^ ^8^ ^]^ However, an excessive mtROS resulting from mETC dysfunction can promote protein unfolding and aggregation, ultimately leading to a compromised and dysfunctional state of the mitochondria.^[^ ^9^ ^]^ These consequences can contribute to a spectrum of diseases and disorders, including neurodegeneration, metabolic syndrome, and aging.^[^ ^10^ ^]^

CI is initially formed by a subcomplex consisting of ≈89 kDa, which includes the core subunit NDUFS3, in conjunction with NDUFS2 and NDUFA5.^[^ ^11^ ^]^ While NDUFS3 itself does not possess direct catalytic activity, it plays a pivotal role in CI assembly. Missense mutations in the NDUFS3 gene have been linked to various diseases, such as Leigh syndrome, and deficiency of NDUFS3 can result in mitochondrial myopathy and optic atrophy.^[^ ^11^, ^12^ ^]^ These prior findings underscore the critical importance of an appropriate structure and composition for the biogenesis and assembly of CI. Such precision is essential to enable the complex to efficiently perform catalytic functions while minimizing potential harm to the mitochondria or the cell.

Serine/arginine‐rich splicing factors (SRSFs) have undergone extensive investigation due to their significant contributions to constitutive splicing and alternative splicing (AS).^[^ ^13^ ^]^ Typically, SRSFs consist of one or two RNA recognition motifs (RRMs) and one serine/arginine‐rich (RS) domain. Among these splicing regulators, SRSF1, serving as the archetype member of the SR protein family,^[^ ^14^ ^]^ has the capability to modulate AS isoform generation by binding to specific RNA sequences and influencing spliceosome activity.^[^ ^15^ ^]^ While SRSF1 is recognized for its involvement in various biological processes and diseases, including the cell cycle, apoptosis, adipocyte differentiation, stress response, cancer, and neurodegeneration,^[^ ^16^ ^]^ its precise function in mature adipocytes still lacks clarity.

In the study, we investigate the role of SRSF1 in adipose tissue by generating adipose‐specific SRSF1 knockout mice. Our analysis revealed that the absence of SRSF1 had a disruptive impact on mitochondrial integrity, leading to a significant whitening of BAT characterized by a reduction in the number of mitochondria. By employing single‐nucleus RNA sequencing, we identify shifts in cell type composition and alterations in differentiation pathways within BAT in the absence of SRSF1. Further investigation reveals the pivotal role of SRSF1 in promoting the inclusion of constitutive exon 6 of Ndufs3, a crucial subunit of mitochondrial complex I. In the absence of SRSF1, exon 6 of Ndufs3 is skipped, resulting in diminished production of functional protein and disrupted mitochondrial function. Notably, the overexpression of native Ndufs3 alleviates mitochondrial defects and restores their function in SRSF1‐knockdown adipocytes. These findings underscore the important role of SRSF1‐mediated splicing of Ndufs3 in mitochondrial homeostasis and brown adipocyte thermogenesis, offering novel insights into the significance of splicing regulation in metabolic processes.

## 2 Results

### 2.1 Inactivation of SRSF1 in Adipose Tissue Triggers the “Whitening” of BAT and Leads to Metabolic Disorders in Mice

**Figure 1:**
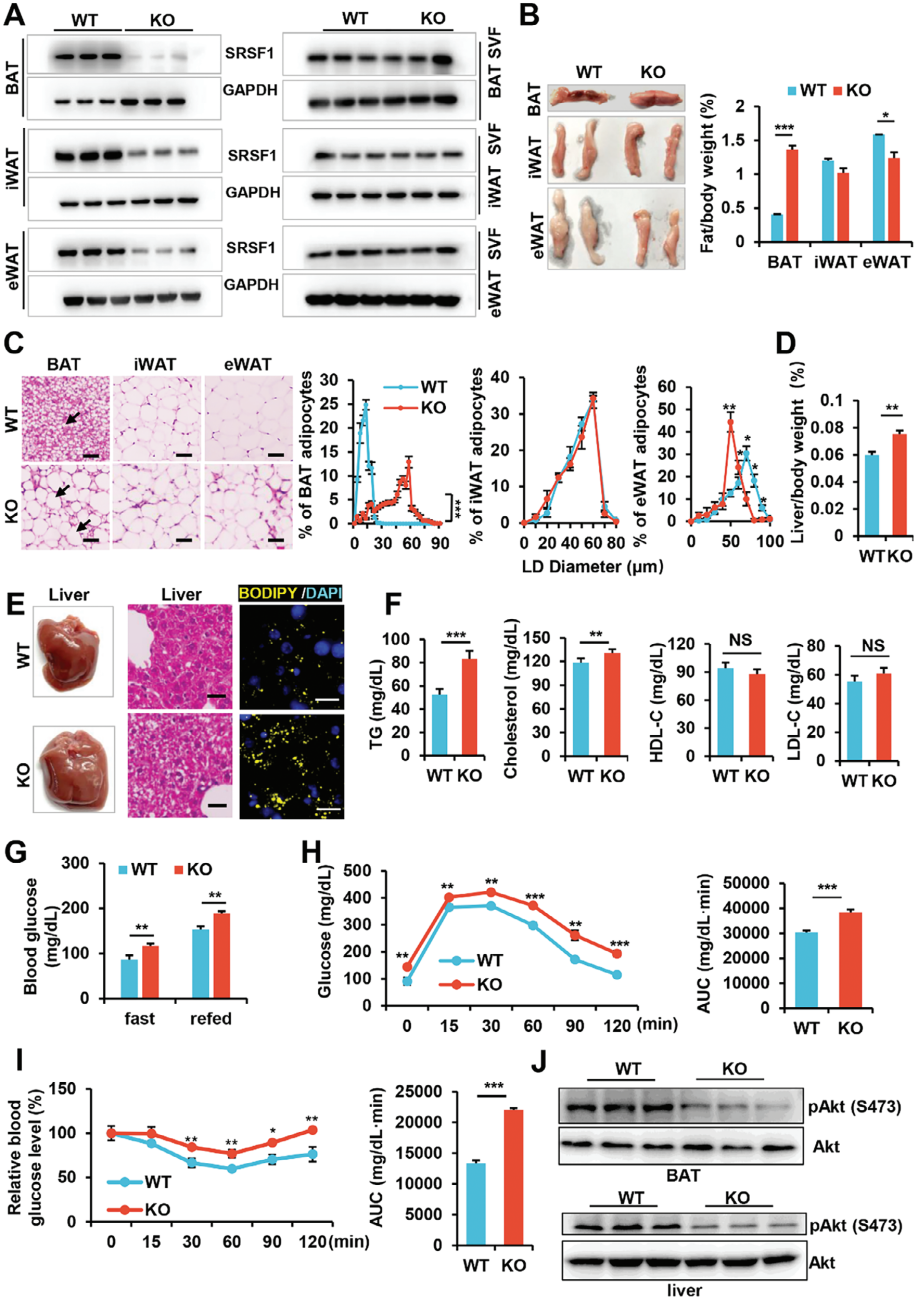
Deletion of SRSF1 in adipose tissue caused whitening of BAT and metabolic disorders in mice. A) Protein levels of SRSF1 were assessed via western blot in the BAT, iWAT, and eWAT of 8‐week‐old WT and KO mice (Left). SRSF1 protein levels were analyzed in the SVF of BAT, iWAT, and eWAT from 8‐week‐old WT and KO mice (Right). B) Representative pictures of BAT, iWAT, eWAT from 8‐week‐old WT and KO mice were shown on the left. The ratio of BAT, iWAT, and eWAT relative to body weight in 8‐week‐old WT and KO mice fed a chow diet was measured and shown on the right graph (n = 13). C) Representative HE images of BAT, iWAT, and eWAT from 8‐week‐old WT and KO mice were shown on the left. The presence of large unilocular lipid droplets (LDs) in BAT was indicated by arrowheads. Scale bar, 50 µm. The LD diameters in BAT, iWAT, and eWAT for both WT and KO mice were measured and presented as the mean ± SEM of 400 cells per sample on the right panel (n = 4 mice per group). D) The ratio of liver relative to body weight of 8‐week‐old WT and KO mice fed a chow diet was measured. E) Representative pictures of liver samples from 8‐week‐old mice were shown on the left. The middle images show the liver sections stained with HE, while the right images show the liver sections stained with BODIPY 493/503. The yellow color represents LDs, and the nuclei are stained with DAPI. The scale bar is 25 µm for the middle panel and 50 µm for the right panel. F) The lipid profile, including plasma levels of TG, cholesterol, HDL‐C, and LDL‐C, was measured from 8‐week‐old WT and KO mice on a chow diet (n = 8). G) Blood glucose levels were measured in 16‐hour‐fasted WT and KO mice at 8 weeks of age on a chow diet (n = 12). H) Blood glucose concentrations were monitored during a GTT (n = 6). The area under the curve (AUC) is depicted in the right graph. I) Blood glucose levels were represented as a percentage of basal glycemia during intraperitoneal ITT in both WT and KO mice. The AUC is shown in the right graph. J) Western blot analysis was conducted to evaluate total Akt and Akt phosphorylation in BAT and liver extracts obtained from 8‐week‐old WT and KO mice that were maintained on a chow diet. ^*^ *p* < 0.05, ^**^ *p* < 0.01, ^***^ *p* < 0.001 by Student's t‐test. Data represent the mean ± SEM.

To investigate the role of SRSF1 in adipose tissue and whole‐body energy metabolism, we generated an adipose‐specific SRSF1‐knockout (KO) mouse model by breeding SRSF1^flox/flox^ mice with transgenic C57BL/6J mice expressing Cre recombinase controlled by the mouse adiponectin (Adipoq) promoter active in mature adipocytes.^[^ ^17^ ^]^ The KO mice carried the Adipoq‐Cre;SRSF1^flox/flox^ genotype, while the SRSF1^flox/flox^ mice served as the wild‐type (WT) control group (Figure S1A, Supporting Information). Confirmation of SRSF1 deletion was achieved through western blot analysis in BAT, inguinal WAT (iWAT), and epididymal WAT (eWAT) (**Figure** **1**A, left panel). The stromal‐vascular fraction (SVF) consists of preadipocytes, endothelial cells, fibroblasts and other cell types within adipose tissue, excluding mature adipocytes. Notably, the retention of SRSF1 in SVF cells emphasizes the specificity of the adipocyte‐targeted SRSF1 deletion (Figure 1A, right panel).

Both KO mice and WT mice exhibited comparable body weights and food intake level when fed a standard chow diet (Figure S1B,C, Supporting Information). However, KO mice displayed a notable increase in BAT mass, a slight decrease in eWAT mass, and no significant change in iWAT mass compared to WT mice (Figure 1C). Interesting, KO mice exhibited a striking whitening effect in BAT, characterized by the accumulation of enlarged lipid droplets (LDs) (Figure 1C). In eWAT, there was variation in adipocyte size, with smaller LDs observed in KO mice compared to WT mice, while no significant difference was observed in iWAT between the two groups (Figure 1C). Additionally, KO mice manifested a pronounced fatty liver phenotype, evidenced by a significant increase in the liver‐to‐body weight ratio (Figure 1D). This lipid droplet accumulation was confirmed through hematoxylin and eosin (HE) staining and BODIPY staining (Figure 1E). The substantial lipid presence in BAT and liver tissues pointed toward a disruption of lipid metabolism in KO mice.

We conducted assessments of the lipid profile by measuring serum levels of triglycerides (TGs), cholesterol, high‐density lipoprotein (HDL), and low‐density lipoprotein (LDL). Our findings revealed elevated levels of TGs and cholesterol in KO mice when compared to their WT counterparts (Figure 1F). However, there was no significant differences in HDL and LDL levels between the two groups. These results substantiate the presence of a lipid metabolism disorder in adipose‐specific SRSF1 deficient mice. Moreover, when we compared fasting and refeeding serum glucose levels, KO mice exhibited significantly higher glucose levels than WT mice fed on a chow diet (Figure 1G). Glucose and insulin tolerance tests (GTTs and ITTs, respectively) unveiled that KO mice had severe glucose intolerance and insulin resistance (Figure 1H,I). In line with the ITT data, the response of insulin on Akt phosphorylation in BAT and liver were diminished in KO mice (Figure 1J). In conclusion, our results demonstrate that SRSF1 deficiency in adipose tissue leads to BAT whitening, hepatic steatosis, hypertriglyceridemia, and insulin resistance

### 2.2 SRSF1 Knockout Mice Exhibited Pronounced Cold Intolerance and Impaired Thermogenesis

**Figure 2:**
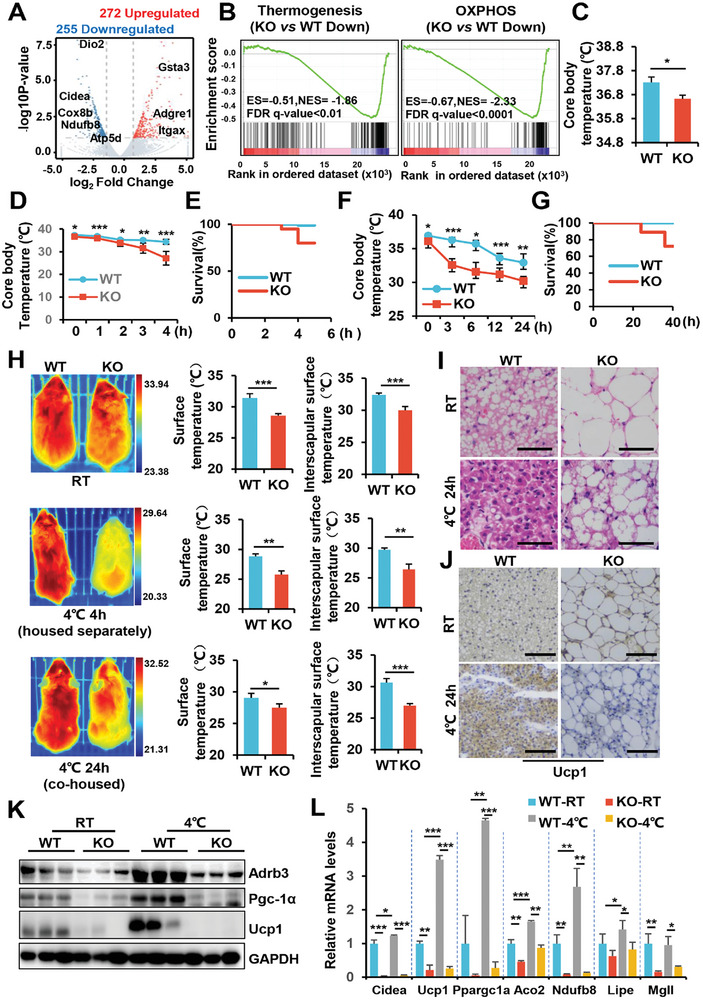
SRSF1 deficiency impairs the thermogenic function of BAT. A)Volcano plot showing DEGs in BAT of both WT and KO mice using RNA‐seq data. Biomarkers relative to thermogenesis and inflammation were highlighted using different colors. B) Gene Set Enrichment Analysis (GSEA) identifying the most significantly downregulated pathways, including thermogenesis and OXPHOS. C) The rectal temperature of 8‐week‐old WT and KO mice was measured at RT (n = 6). D) The rectal temperature of 8‐week‐old mice, individually housed, was measured after subjecting them to cold exposure (4 °C) (n = 8). No food was provided to the mice during this period. E) Survival curves was plotted for WT and KO mice exposed to cold (4 °C), with the mice housed individually in separate cages without food (n = 16). F)The core body temperature of 8‐week‐old WT and KO mice was measured under cold stimulation conditions (4 °C). During this period (24 h), mice were co‐housed together and provided with food (n = 8). G) Survival curves were also plotted for mice co‐housed and exposed to cold for 48 h (n = 20). H)Thermal images, along with the surface temperature and iBAT temperature of 8‐week‐old WT and KO mice, were displayed under different conditions. In the top panel, the mice were kept at RT. In the middle panel, mice were individually housed at 4 °C for 4 h without food. In the bottom panel, WT and KO mice were co‐housed at 4 °C for 24 h with food. The color scale represents the temperature range, with blue indicating cold and red indicating hot. I,J) Representative images of HE staining (I) and Ucp1 immunohistochemistry (J) of BAT from mice housed at RT or 4 °C for 24 h are shown. Scale bar: 50 µm (I), 100 µm (J). K,L) The protein levels (K) or mRNA levels (L) of thermogenic genes in BAT of WT mice and KO mice were measured after exposing the mice to cold temperature (4 °C). WT and KO mice were co‐housed at 4 °C for 24 h (n = 4), and the results were compared to mice housed at RT. The q‐PCR data analysis involved measuring amplicon abundance via the 2^−ΔΔCT^ method, normalized to β‐Actin. Each gene's value in WT‐RT was set as 1 to assess the relative mRNA levels of target genes across the KO‐RT, WT‐4 °C, and KO‐4 °C groups. ^*^ *p* < 0.05, ^**^ *p* < 0.01, ^***^ *p* < 0.001. Data represent the mean ± SEM.

Considering the notable increase in lipid accumulation and the pronounced whitening phenotype observed in the interscapular BAT of KO mice, we decided to investigate how SRSF1 deficiency affects BAT function. To accomplish this, we conducted high‐throughput RNA‐seq on the BAT samples collected from 8‐week‐old WT and KO mice. Our analysis clearly revealed that key thermogenic marker genes, including Dio2, Cidea, and Cox8b, were downregulated in KO mice, while markers associated with inflammation, such as Itgax and Adgre1, were upregulated (**Figure** **2**A). Furthermore, gene set enrichment analysis (GSEA) indicated that the downregulated genes in the BAT of KO mice were predominantly enriched in pathways related to thermogenesis and OXPHOS (Figure 2B). Correspondingly, KO mice exhibited a significant decrease in their core body temperature during both feeding and fasting periods when kept at room temperature (RT) (Figure 2C; Figure S2A, Supporting Information).

To further explore the impact of SRSF1 deficiency on adaptive thermogenesis, we conducted cold stimulation experiments for varying durations on mice. For acute cold exposure without food, individual mice were placed in separate cages at 4 °C. Notably, KO mice experienced a rapid decline in core body temperatures over time, ultimately leading to mortality after 3 h (Figure 2D,E). In contrast, WT mice maintained core body temperatures above 30 °C and exhibited no mortality under the same conditions (Figure 2D,E). When provided food during 6 h cold exposure (housed separately) and extended periods of cold exposure (co‐housed), KO mice consistently displayed lower rectal temperatures than WT mice (Figure S2B, Supporting Information; Figure 2F). Furthermore, while KO mice survived the 24 h stimulation period, they exhibited a higher mortality rate after the 24 h period (Figure 2G). Thermal imaging analysis unveiled that the surface temperatures and brown fat temperatures in the interscapular region of KO mice were reduced when they were housed at RT compared to WT mice. Furthermore, this reduction in temperature became even more pronounced in KO mice at 4°, particularly when KO mice were housed individually (Figure 2H).

HE staining revealed that cold exposure led to a reduction in fat droplet size in the BAT of WT mice, indicating enhanced lipolysis and thermogenesis. In contrast, KO mice did not exhibit a significant reduction in lipid accumulation within BAT (Figure 2I). Ucp1, a mitochondrial transmembrane protein, functions by decoupling OXPHOS from ATP production.^[^ ^18^ ^]^ Mice lacking Ucp1 are susceptible to developing hypothermia when exposed to sudden cold conditions.^[^ ^19^ ^]^ The tightly controlled expression of Ucp1 in brown and beige adipocytes established Ucp1 as a pivotal indicator of thermogenesis.^[^ ^20^ ^]^ Immunohistochemical analysis further revealed reduced immunoreactivity of Ucp1 in the BAT of KO mice following cold exposure (Figure 2J). Additionally, western blot analysis demonstrated a significant increase in the levels of thermogenic proteins, including Adrb3, Pgc‐1α, and Ucp1 in the BAT of WT mice after exposure to cold stimulation. In contrast, KO mice did not exhibit a significant induction of these proteins following cold stimulation, and their expression remained lower in KO mice even when kept at RT, compared to WT mice (Figure 2K). Furthermore, the mRNA levels of thermogenic genes (Cidea, Ucp1, Ppargc1a), genes involved in OXPHOS (Aco2 and Ndufb8), as well as the lipolysis genes (Lipe and Mgll), were found to be decreased in the BAT of KO mice. Moreover, the expression of these genes was not stimulated in response to cold exposure in KO mice compared to WT mice (Figure 2L). Collectively, these findings strongly support the conclusion that the reduced expression of thermogenic genes led to impaired thermogenesis in KO mice.

To further substantiate this, we isolated primary brown adipocytes from the BAT of WT and KO mice at 1 day old and cultured them in differentiation medium until maturity. As shown, the brown adipocytes lacking SRSF1 exhibited significantly lower levels of thermogenic‐related proteins (Figure S2C, Supporting Information). Even treatment with Forskolin (FSK,) a cAMP‐PKA activator, failed to restore their expression (Figure S2D, Supporting Information). This points to dysfunction in brown adipocytes as the primary cause of impaired BAT thermogenesis in the KO mice.

### 2.3 SRSF1 Deficiency Impairs β‐Adrenergic Agonist‐Induced Thermogenesis in BAT

**Figure 3:**
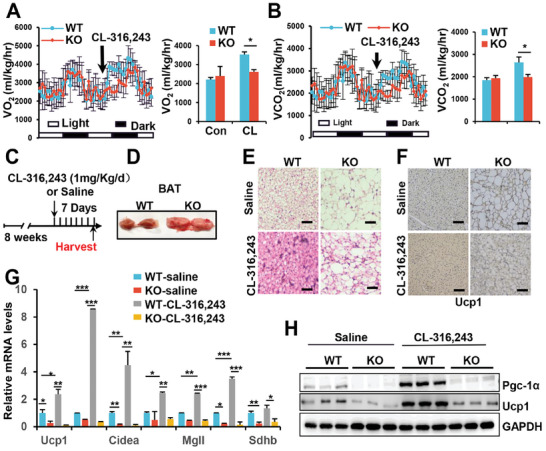
SRSF1 deficiency impairs β3‐adrenergic agonist‐induced thermogenesis of BAT. A,B) O_2_ consumption (A) or CO_2_ production (B) were recorded in WT and KO mice during a 48 h light‐dark cycle, both before and after an injection of CL‐312643 at a dosage of 1 mg kg^−1^. O_2_ consumption or CO_2_ production in WT and KO mice was also measured before and after 1 h of CL‐312643 treatment, and the results are shown on the right. C) A schematic design of the CL‐312643 treatment regimen is presented. Mice were kept on a standard diet for 8 weeks, followed by intraperitoneal injections of either saline or CL‐316243 (1 mg kg^−1^) in both WT and KO mice for 7 consecutive days. The experiments were conducted on the 8th day, after a 7‐day injection period. D) Representative images of BAT in both KO and WT mice were obtained after continuous injection of CL‐316243 for 7 days. E,F). Representative images of HE staining (E) and Ucp1 immunohistochemistry (F) of BAT from mice treated as described in (C). Scale bar: 50 µm (E); 100 µm (F). G) The relative expression of thermogenic genes in BAT was assessed in mice, as detailed in (C) (n = 3), employing the method described in Figure 2L. H) The expression of thermogenic proteins in BAT was evaluated in mice treated as described in (C). ^*^ *p* < 0.05, ^**^ *p* < 0.01, ^***^ *p* < 0.001. Data represent the mean ± SEM.

CL‐316243, a selective β3‐adrenergic receptor agonist, is commonly used to mimic cold exposure and activate BAT activity.^[^ ^21^ ^]^ To investigate the significant role of SRSF1 in systemic metabolism, we conducted a study employing indirect calorimetry to examine the acute metabolic response of WT and KO mice to adrenergic agonists. Interestingly, there were no differences in oxygen consumption and carbon dioxide production between KO and WT mice without CL‐316243 stimulation, suggesting that the reduced OXPHOS and thermogenic capacity of KO mice did not significantly impact whole‐body metabolism in a resting state (**Figure** **3**A,B). However, the administration of CL‐316243 resulted in a substantial increase in oxygen consumption and carbon dioxide production in WT mice, while having a minimal effect on KO mice (Figure 3A,B). This finding indicated that SRSF1 is essential for the metabolic response to CL‐316243.

Research has shown that adrenal hormone stimulation of BAT promotes tissue remodeling by enhancing enhanced mitochondrial biogenesis and Ucp1 expression.^[^ ^22^ ^]^ To investigate whether SRSF1 is essential for BAT remodeling in response to adrenergic stimulation, mice were injected with CL‐316243 or saline (control) for 7 days (Figure 3C). After 7‐days treatment with CL‐316243, BAT from KO mice still exhibited a whitening effect compared to BAT from WT mice (Figure 3D). HE staining indicated a significant reduction in the size of BAT lipid droplets in WT mice, while the BAT of KO mice showed excessive accumulation of lipid droplets (Figure 3E). Immunohistochemical staining further demonstrated a significant increase in Ucp1 expression in WT mice treated with CL‐316243, while this induction was compromised in KO mice (Figure 3F). Moreover, the administration of CL‐316243 in WT mice resulted in a considerable upregulation of genes related to thermogenesis, lipolysis, and OXPHOS in BAT, whereas this response was significantly impaired in the BAT of KO mice (Figure 3G). Western blot analysis provided further support for these findings, revealing a significant increase in the expression of thermogenic proteins in the BAT of WT mice following agonist treatment. However, this induction was markedly compromised in the BAT of KO mice (Figure 3H).

### 2.4 SRSF1 Regulates the Browning of WAT Following Treatment with a β3‐Adrenergic Receptor Agonist

In addition to its role in regulating BAT thermogenesis, the stimulation of β3‐adrenergic receptor agonist can also induce the browning of WAT.^[^ ^2^ ^]^ Therefore, we investigated the potential of CL‐316243 to induce browning of WAT in both WT and KO mice. Our experimental approach involved treating both KO and WT mice with CL‐316243 for 7 days. Interestingly, CL‐316243 treatment resulted in a significant increase in multilocular adipocytes and the characteristic browning in the iWAT and eWAT of WT mice. However, the effect of CL‐316243 on adipocytes in KO mice was significantly reduced (Figure S3A,B, Supporting Information). Furthermore, treatment of with CL‐316243 led to enhanced expression of Ucp1 proteins in both iWAT and eWAT from WT mice, while Ucp1 induction in KO mice was noticeably reduced (Figure S3A,B, Supporting Information). Additionally, the induction of both thermogenic proteins and thermogenic gene expression was significantly impaired in KO mice treated with CL‐316243 (Figure S3C–F, Supporting Information). In conclusion, our results demonstrated that SRSF1 deficiency hindered the induction of beige adipocytes mediated by adrenergic receptor agonists in WAT, highlighting the important role of SRSF1 in regulating the browning process and the thermogenic response of adipocytes in WAT.

### 2.5 Single Nucleus RNA Seq (snRNA‐seq) Unveils Distinctive Cell Types in the BAT of KO Mice

**Figure 4:**
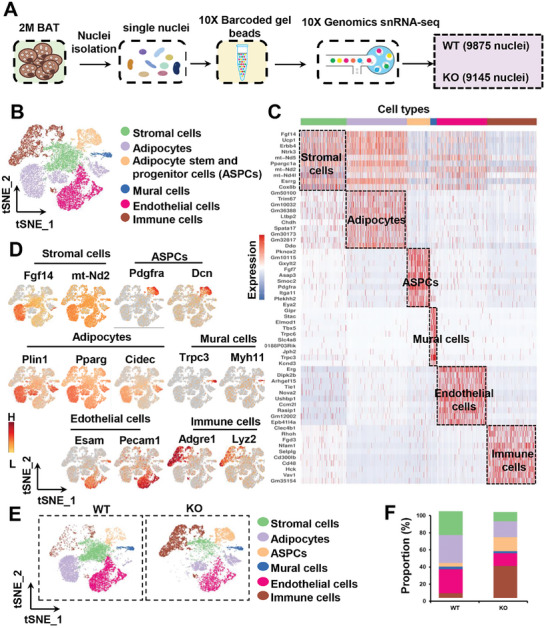
snRNA‐seq uncovers cellular population heterogeneity in BAT between WT and KO mice. A) Overview of the experimental workflow: BAT was isolated from both WT and KO mice, dissociated into single‐nucleus suspensions, followed by snRNA‐seq and subsequent bioinformatic analyses. B) t‐SNE plot illustrates the distribution of various cell types within BAT. C) The heatmap displays the top 10 DEGs within each cell type of BAT, chosen according to the gene‐diff criteria. D) Expression patterns of representative marker genes are depicted for each cell type. The scale bar represents gene expression levels, with deeper red shades indicating higher expression (denoted as H) and lighter colors indicating lower expression (denoted as L). E) Separate t‐SNE plots are generated for the WT and KO datasets to explore potential differences in cellular distribution. F) Comparative analysis reveals the proportion of different cell types between BAT samples from WT and KO mice.

To investigate cell population heterogeneity and explore the molecular and pathway characteristics linked to thermogenic function, we conducted snRNA‐seq analysis on BAT samples obtained from 8‐week‐old WT and KO mice. In total, we collected 24341 nuclei, and after quality control we further analyzed data from 19020 single nuclei (**Figure** **4**A). The initial clustering and marker gene annotation identified 13 major cell populations (Figure S4A, Supporting Information). Based on the representative markers, we classified these clusters into 6 distinct cell types, namely stromal cells (Fgf14, mt‐Nd2; 19.3%), adipocytes (Plin1, Pparg, Cidec; 25.6%), adipose stem and progenitor cells (ASPCs) (Pdgfra, Dcn; 10.0%), mural cells (Trpc3, Myh11; 2.7%), endothelial cells (Esam, Pecam1; 21.4%), and immune cells (Adgre1, Lyz2; 21.0%) (Figure 4B–D).

Interestingly, the adipocyte population in KO mice exhibited a notable shift in its clustering distribution on the t‐SNE plot, indicating distinct molecular and functional features of these adipocytes (Figure 4E). Analysis of WT and KO cell proportion in each cluster showed a decrease in the frequencies of stromal cells, adipocytes, and endothelial cells in the BAT of KO mice. The KO mice exhibited a dramatic increase in the presence of ASPCs and immune cells. No significant differences were observed in the proportion of mural cells in the KO mice (Figure 4F).

Examination of the top 20 differentially expressed genes (DEGs) revealed extensive downregulation of genes involved in thermogenesis (Ucp1, Cidea, Dio2, Ppargc1a, and Elovl6) in KO mice compared to WT mice (Figure S4B, Supporting Information). In contrast, inflammation‐related genes such as Alcam, Dock2 and Adgre1 were upregulated in KO mice (Figure S4B, Supporting Information). We annotated the DEGs identified in the comparison between WT and KO mice using the KEGG pathway analysis. This analysis revealed that the upregulated pathways were primarily associated with the inflammatory response, whereas the predominantly downregulated pathways were related to thermogenesis and OXPHOS (Figure S4C, Supporting Information). Overall, these findings highlight the significant impact of SRSF1 on the cellular landscape and molecular pathways associated with BAT function.

### 2.6 A Distinctive Subcluster of Brown Adipocyte, Designated as BA‐3, Demonstrates Metabolic Dysfunction in the BAT of KO Mice

**Figure 5:**
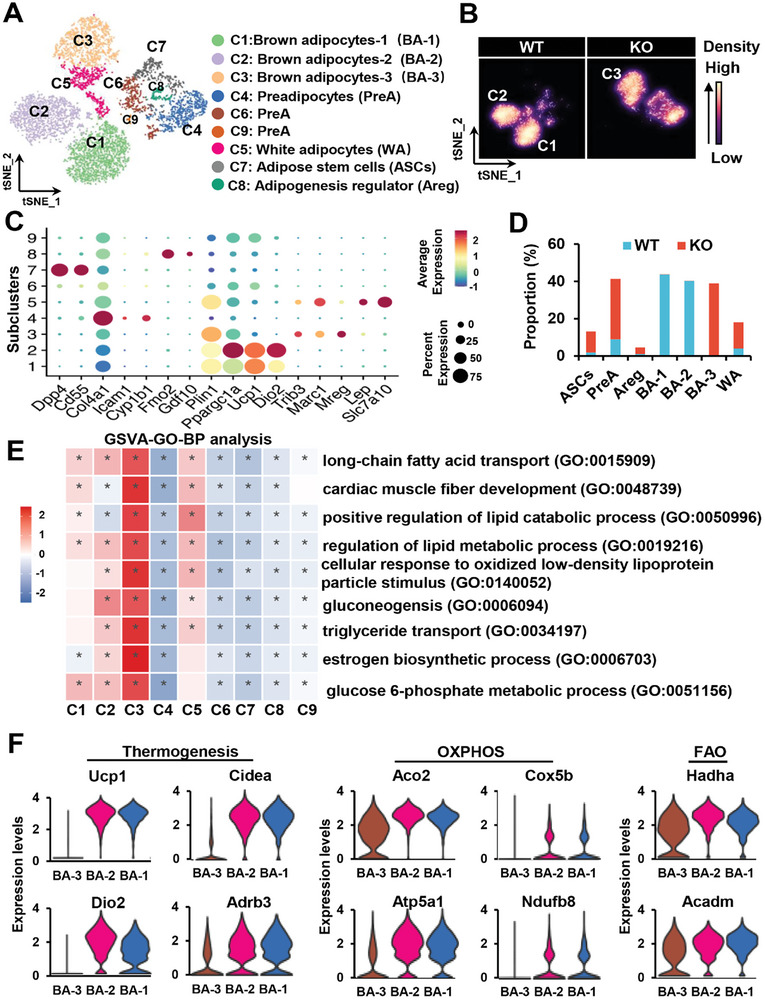
Identification of a distinct subpopulation of brown adipocytes with impaired metabolism in SRSF1‐deficient BAT. A) t‐SNE analysis reveals the existence of nine distinct subclusters of adipocytes. B) The density plot demonstrates differences in the distribution subclusters between WT and KO samples. C) The dot plot displays the expression levels of selected marker genes for each subcluster, with dot size indicating the percentage of cells expressing a specific gene and color intensity representing the average expression level. D) A stacked bar plot provides a visual representation of the proportion of cells from WT or KO samples within each adipocyte subcluster. E) GSVA‐GO analysis of biological processes highlights the functional characteristics and distinctions of BA‐3 in comparison to other adipocyte subtypes. F) Violin plots depict the expression levels of genes related to thermogenesis, OXPHOS, and FAO among BA‐1, BA‐2, and BA‐3.

To analyze the population of adipocytes and adipocyte stem/progenitor cells, we conducted unsupervised clustering in both WT and KO mice. As a result, we identified a total of nine unique subclusters, labeled as C1 to C9 (**Figure** **5**A). When the t‐SNE plot was segregated, a significant difference in the distribution of brown adipocytes (C1‐C3) was observed (Figure 5B). This disparity indicates a potential alteration in the function of brown adipocytes in KO mice compared to WT mice. As illustrated in Figure 5C, subclusters (C1, C2, C3) characterized by the marker Plin1 were identified as mature brown adipocytes. C1 and C2, which displayed relatively higher expression of Ppargc1a, Ucp1, and Dio2, were predominantly observed in WT mice and designated as brown adipocytes‐1 and brown adipocytes‐2 (BA‐1 and BA‐2) (Figure 5B,C). Conversely, C3 was exclusively present in KO mice and exhibited a unique gene expression pattern, with notable upregulation of stress‐induced genes such as Trib3, Marc1, and Mreg (Figure 5B,C). Therefore, this cluster was labeled as BA‐3. C5, displaying elevated expression of Lep and Slc7a10, well‐known markers for white adipocytes, were identified as white adipocytes (WA). Adipose stem cell markers, Dpp4 and Cd55, were exclusively expressed in C7. C8, characterized by high levels of Fmo2 and Gdf10, was annotated as the adipogenesis regulator (Areg) cluster. Finally, C4, C6, and C9, characterized by dominant expression of Col4a1, Icam1, or Cyp1b1, were identified as preadipocytes (PreA) clusters. (Figure 5A,B). Additionally, the KO mice showed a reduction in the number of brown adipocytes, accompanied by an increase in the number of WA, ASCs, Pre‐A, and Areg cells (Figure 5D).

Gene Ontology enrichment analysis (GO) using gene set variation analysis (GSVA) revealed that BA‐3 primarily participated in the transport of TGs and fatty acids, as well as in the cellular responses to oxidative stress and inflammation (Figure 5E). Notably, a substantial reduction in the expression of genes associated with thermogenesis (Ucp1, Cidea, Dio2, Adrb3), OXPHOS (Aco2, Cox5b, Atp5a1, Ndufb8), and fatty acid oxidation (FAO) (Hadha, Acadm) was observed in BA‐3 compared with the BA‐1 and BA‐2 (Figure 5F). In contrast, BA‐1 primarily engaged in amino acid and acetyl‐CoA metabolism, along with hormone response (Figure S5A, Supporting Information). BA‐1 exhibited predominant expression of genes related to lipogenesis (Acly, Fasn, Acaca), indicating its primary role in lipid synthesis and energy storage within BAT (Figure S5B, Supporting Information). BA‐2 was mainly associated with FAO (Figure S5A, Supporting Information). BA‐2 displayed significantly higher expression of genes linked to thermogenesis (Dio2) and FAO (Hadha, Hadhb, Cpt1b, Ppara) compared to BA‐1 (Figure S5C,D, Supporting Information). These findings strongly suggest that BA‐2 plays a pivotal role in adipocyte thermogenesis.

The results indicate that the deficiency of SRSF1 contributes to alternations in cell type composition within BAT, transitioning from functional brown adipocytes in WT mice to impaired brown adipocytes and white adipocytes in KO mice.

### 2.7 Pseudotime Analysis Reveals Distinct Differentiation Trajectories and Mitochondrial Dysfunction in brown Adipocytes Following SRSF1 Deficiency

Next, we wanted to utilize pseudotime analysis to reconstruct the trajectories of brown adipocytes and evaluate the cellular dysfunction arising from SRSF1 deletion. To ensure accurate trajectory analysis of brown cell differentiation, our approach involves excluding the white adipose cell population.

**Figure 6:**
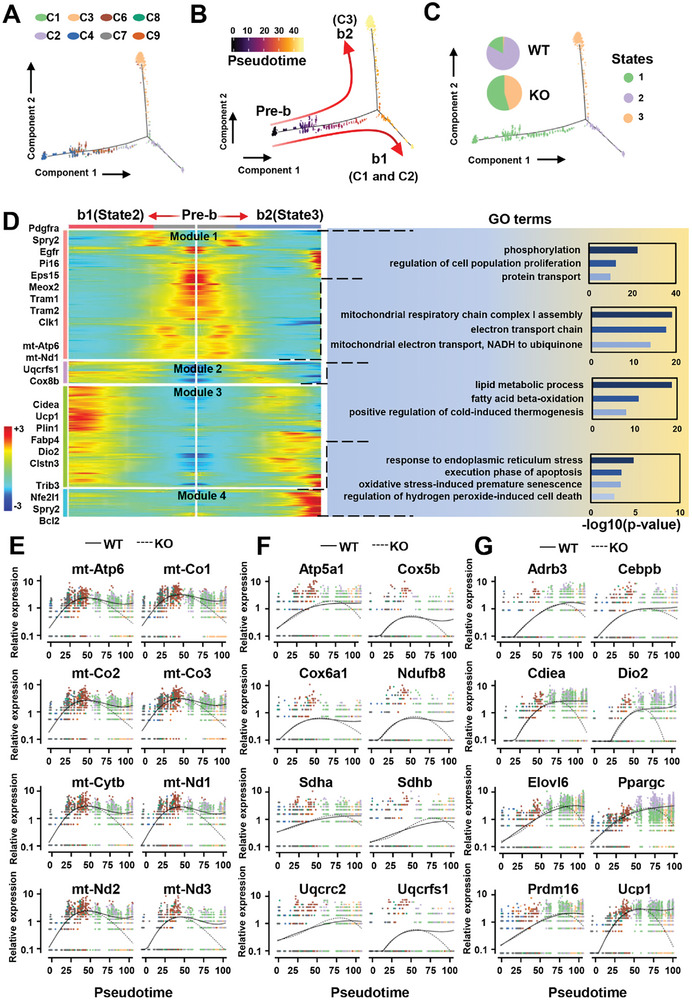
Pseudotime analysis reveals abnormal differentiation pathways and mitochondria dysfunction within BAT of KO mice. A) The pseudotime trajectory map displays the identities of the subclusters. B) Pseudotime progression is represented from black to light yellow. C) The map illustrates the relationship of all subclusters to the three developmental stages (states) based on gene expression. The composition of each state in WT and KO samples is shown in a pie chart, with each section divided equally according to the pseudotime of the cells. D) A heatmap arranges all genes with dynamic expression patterns along pseudotime (left). Significantly enriched GO terms for each module are presented on the right. E–G). Scatterplot graphs depict the expression dynamics of the selected genes along pseudotime, providing a detailed view at the resolution of individual cells. Expression levels of genes related to mitochondrial DNA replication (E), OXPHOS (F), and thermogenesis (G) are plotted against pseudotime. Solid lines represent the b1 branch (WT), while dashed lines represented the b2 branch (KO).

Monocle2 organized ASCs (C7), PreA (C4, C6, and C9), Areg (C8), BA‐1 (C1), BA‐2 (C2), and BA‐3 (C3) along a shared trajectory that bifurcated into two distinct branches (**Figure** **6**A). ASCs and PreA cells were primarily located at the early part of the pre‐branch (Pre‐b), while BA‐1 and BA‐2 cells resided at or near the terminus of the primary branch b1. Likewise, BA‐3 cells occupied at or near the terminus of the main branch b2 (Figure 6A,B). Furthermore, all cells were categorized into three states. State 1 consisted of cells from both WT and KO mice, whereas state 2 and state 3 were predominately occupied by cells from WT and KO mice, respectively (Figure 6C). This observation suggests that brown adipocytes in WT and KO mice display distinct molecular characteristics and functional properties within the analyzed trajectory.

Upon close examination of the trajectory, we identified genes that exhibited significant differential expression over pseudotime. These genes were then categorized into four modules based on their temporal expression patterns, as depicted in the heatmap in Figure 6D. Specifically, genes in Module 1 (e.g., Clk1, Pi16, Tram1) displayed high expression levels at the initial Pre‐b stage, with their expression gradually decreasing along pseudotime. These genes were found to be enriched in terms related to phosphorylation, cellular proliferation, and protein transport, indicating their role in precursor adipocytes. Interestingly, both WT and KO mice exhibited similar gene expression patterns, suggesting no significant differences in the characteristics of these precursor adipocytes (Figure 6D).

Module 2, in contrast, exhibited a significant enrichment of genes associated with mitochondrial functions, including mt‐Atp6, mt‐Nd1, Uqcrfs1, and Cox8b. The expression of these genes displayed a gradual increase along the b1 branch, without notable accumulation at the end of the b2 branch (Figure 6D–F). This observation strongly indicated impaired mitochondrial function in cells from KO. Module 3 focused on thermogenic genes such as Ucp1, Ppargc1a and Dio2, which are typically highly expressed in mature brown adipocytes. The expression of these genes progressively increased as pseudotime advanced, reaching peak levels during the late maturation stage in cells situated at the end of the b1 branch. Conversely, cells at the end of the b2 branch exhibited decreased expression of thermogenesis‐related genes in Module 3, providing clear evidence of impaired BAT function in KO mice (Figure 6D,G). Module 4 was predominantly localized in cells at the terminus of the b2 branch. Functional characterization of Module 4 through GO analysis indicated its participation in pathways related to oxidative stress response and apoptosis (Figure 6D). These findings strongly suggested a significant stress response in cells from KO mice.

### 2.8 SRSF1 Deletion Compromises Mitochondrial Integrity and Triggers an Excessive mtROS Production

Pseudotime analysis revealed mitochondrial dysfunction in brown adipocytes of KO mice. The results of GO analysis for BA‐1, BA‐ 2, and BA‐3 further emphasized the pronounced mitochondrial dysfunction in cells from BA‐3 compared to BA‐1 and BA‐2 (Figure S6, Supporting Information). Based on these findings, we conclude that the compromised function of adipocytes in KO mice can be primarily attributed to mitochondrial dysfunction.

**Figure 7:**
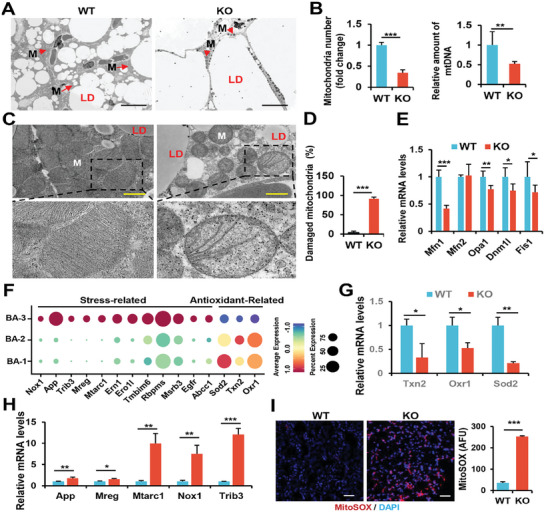
Deletion of SRSF1 disrupted mitochondrial homeostasis, leading to accumulated mtROS. A) Transmission electron microscopy (TEM) images of BAT from 8‐week‐old WT and KO mice are shown at low magnification. Lipid droplets are labeled as “LD”, and mitochondria are indicated as “M” with arrows. Scale bar, 10 µm. B) Comparison of mitochondrial numbers in the BAT of KO and WT mice (left). Measurement of the relative content of mtDNA in the BAT of these mice (right). C) TEM images of BAT at high magnification, highlighting mitochondria. Scale bar, 1 µm. D) Presentation of the percentage of damaged mitochondria in both groups. E) Analysis of mitochondrial fusion and fission genes in BAT samples from 8‐week‐old WT and KO mice using q‐PCR. F) A dot plot illustrates the expression of stress‐related and antioxidant‐related genes in BA‐1, BA‐2, and BA‐3 subpopulations. G,H) q‐PCR analysis of antioxidant genes (G) and stress‐related genes (H) in BAT samples from the same mice mentioned above. H) Measurement of mtROS levels in BAT samples from 8‐week‐old WT and KO mice using MitoSOX (n = 3). Data are presented as arbitrary fluorescence units (AFU). ^*^ *p* < 0.05, ^**^ *p* < 0.01, ^***^ *p* < 0.001. Data represent the mean ± SEM.

We then conducted transmission electron microscopy on BAT samples from both WT and KO mice. Our findings unveiled severe mitochondrial degeneration in brown adipocytes of KO mice, characterized by a transformation into small dot‐like structure, accompanied by the accumulation of large‐sized lipid droplets, in stark contrast to the control group (**Figure** **7**A). Additionally, we observed a significant reduction in both mitochondrial abundance and mtDNA copy number (Figure 7B). At high magnifications, mitochondria in WT mice displayed a well‐preserved inner membrane structure with neatly aligned cristae. Conversely, cells from KO mice exhibited severely disrupted inner mitochondrial membranes, along with a higher percentage of mitochondria containing fractured inner membranes (Figure 7C,D). Additionally, we assessed the expression of key genes involved in mitochondrial membrane fusion and division, including Mfn1, Mfn2, Opa1, Dnm1l, and Fis1. Notably, we observed lower expression levels of these genes in KO mice compared to WT mice, with the exception of Mfn2 (Figure 7E). In conclusion, our results strongly indicate that deficiency of SRSF1 in BAT leads to compromised mitochondrial integrity.

Furthermore, the elevated expression of stress‐related genes and the decreased expression of antioxidant genes was observed in BA‐3 when compared to BA‐1 and BA‐2 (Figure 7F), which was consistent with the phenotype of disrupted mitochondria in brown adipocytes of KO mice. Comparable alterations in gene expression were also evident in the BAT of KO mice (Figure 7G,H). To explore whether SRSF1 deficiency led to elevated mtROS production, we assessed mitochondrial oxidative stress levels using MitoSOX, a probe specifically designed for detecting mitochondrial superoxide. Our analysis revealed a pronounced accumulation of mitochondrial superoxide in the BAT of KO mice (Figure 7I). These findings emphasized the crucial role of SRSF1 in preserving mitochondrial integrity and function in brown adipocytes.

### 2.9 SRSF1 Regulates the Inclusion of Constitutive exon 6 of Ndufs3, Promoting the Production of its Functional Protein in BAT

**Figure 8:**
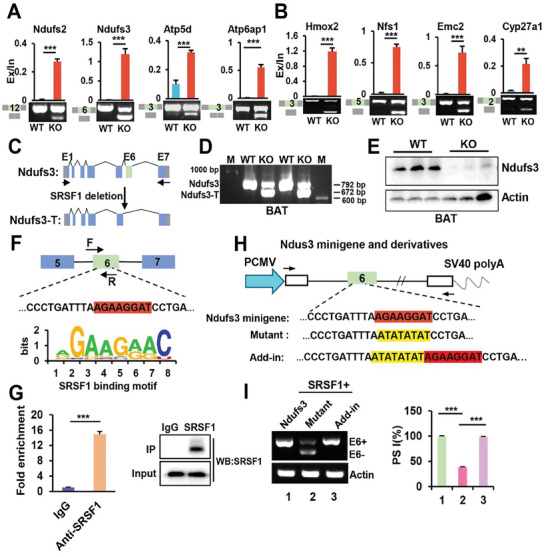
SRSF1 plays a key role in regulating the inclusion of constitutive exon 6 within Ndufs3 pre‐mRNA. A,B) Validation of representative exon inclusion or exclusion events influenced by SRSF1 was conducted through RT‐PCR. The regulated exon, identified with its exon number, is depicted in the green box. The exclusion/inclusion (Ex/In) ratios of RNA products are quantified (n = 3). C) A schematic diagram illustrating the Ndufs3 and Ndufs3‐T isoforms, with or without exon 6. Note that SRSF1 deletion led to the production of Ndufs3‐T. D) Analysis of the expression pattern of Ndufs3 and Ndufs3‐T isoforms in BAT samples collected from WT and KO mice using RT‐PCR analysis. E) Western blot analysis conducted on BAT samples obtained from WT and KO mice using anti‐Ndufs3 antibodies. F) Schematic diagrams illustrating the potential binding sites for SRSF1 on exon 6 of Ndufs3, indicated by the red‐marked sequence. The binding motifs of SRSF1, predicted by the RBP Suite website, are displayed at the bottom. G) q‐PCR analysis performed on Ndufs3 pre‐mRNA in samples of immunoprecipitation (IP) using anti‐SRSF1 antibodies or IgG control (left) in brown adipocytes. The western blot analysis shows SRSF1 protein levels in whole cell extracts (input) and post‐IP using the anti‐SRSF1 antibodies (right). H) Diagrams of the Nduf3 minigene construct and two of its derivatives are presented. The sequence within the exon6, highlighted in red, have been substituted with the sequences marked in yellow in the mutation minigene. I) Brown adipocytes were co‐transfected with the SRSF1‐overexpressing plasmid and either the Ndufs3 minigene or derivatives. In vitro splicing analysis of Ndufs3 E6 was performed using RT‐PCR, and the resulting Percent Spliced In (PSI) values were presented on the right graph. PSI = Inclusion/(Inclusion + Exclusion). ^*^ *p* < 0.05, ^**^ *p* < 0.01, ^***^ *p*,0.001. Data represent the mean ± SEM.

To elucidate the mechanism underlying the impact of SRSF1 deficiency on mitochondria dysfunction in brown adipocytes, we analyzed the RNA‐seq data available to identify potential splicing events regulated by SRSF1 in BAT of KO mice compared with controls. As shown in **Figure** **8**A,B, the absence of SRSF1 resulted in significant exon skipping in genes involved in the assembly of mitochondrial complexes, such as Ndufs2, Ndufs3, Atp5d and Atp6ap1, as well as genes related to mitochondrial function, including hmox2, Nfs1, Emc2, and Cyp27a1. Interestingly, we found that the regulated exons of Ndufs2, Ndufs3, Hmox2, and Emc2 are constitutively spliced (Figure S7, Supporting Information). This suggests that SRSF1 plays a role in regulating the splicing of these constitutive exons in the BAT.

In this study, we focused on examining the Ndufs3 gene for two primary reasons: its established role in mitochondrial function and the notable impact observed due to SRSF1 deficiency (Figure 8A). Our findings revealed that the deletion of SRSF1 caused exon 6 skipping from Ndufs3 pre‐mRNA, resulting in the formation of a truncated isoform (referred to as Ndufs3‐T) (Figure 8C). Although the presence of Ndufs3‐T mRNA in the BAT of KO mice was confirmed through RT‐PCR analysis (Figure 8D), western blot analysis revealed a significant decrease in the levels of Ndufs3 protein in the BAT of KO mice. The protein isoform of Ndufs3‐T was not detected in the KO group, which suggests potential instability of the Ndufs3‐T mRNA caused by the omission of constitutive exon 6 (Figure 8E).

To investigate the mechanism by which SRSF1 regulates the inclusion of exon 6 in Ndufs3, we first utilized the RBP Suite prediction website to determine the potential binding motif for SRSF1 in the regions of Ndufs3 spanning exon 5 to exon 7. The motif analysis conducted on SRSF1‐activated AS events showed a significant enrichment of purine‐rich sequences, particularly AGAAGGAC. In fragment exon 6 of the Ndufs3 gene, we identified a similar sequence, AGAAGGAT, which could potentially serve as a binding site for SRSF1 (Figure 8F). Subsequently, we performed RNA immunoprecipitation (RIP) experiments in brown adipocytes and confirmed the presence of SRSF1 bound to exon 6 of Ndufs3 pre‐mRNA (Figures 8G). To further investigate the association between the binding of SRSF1 to exon 6 and exon inclusion, we created a minigene reporter plasmid, which contained a genomic DNA fragment of Ndufs3 exons 5–7 (Figure 8H). When brown adipocytes were co‐transfected with the minigene and SRSF1‐expessing plasmids, Ndufs3 exon 6 was completely included (Figure 8I, lane 1). We then generated a mutation minigene by replacing AGAAGGAT with AT‐rich sequences in the exon 6. Brown adipocytes transfected with the Ndufs3 mutant showed a significant reduction in exon 6 inclusion (Figure 8I, lane 2). Interestingly, when the SRSF1 binding sequence was restored in the Add‐in minigene, exon 6 skipping was effectively suppressed in these cells (Figure 8I, lane 3 compared with lanes 1, 2). These findings suggested that the binding of SRSF1 is necessary for inclusion of Ndufs3 exon 6.

### 2.10 SRSF1 Preserves Mitochondrial Function by Controlling the Inclusion of exon 6 in Ndufs3 in Primary Brown Adipocytes

Given that Ndufs3 is a constituent of mitochondrial complex I, responsible for electron transport and the generation of mtROS, our study aimed to investigate the impact of Ndufs3 deficiency on mitochondrial function. To achieve this, we designed and employed two siRNAs, namely si‐Ndufs3‐1 and si‐Ndufs3‐2, to knock down Ndufs3. The efficiency of this knockdown was confirmed through qPCR conducted in brown adipocytes (Figure S8A, Supporting Information).

**Figure 9:**
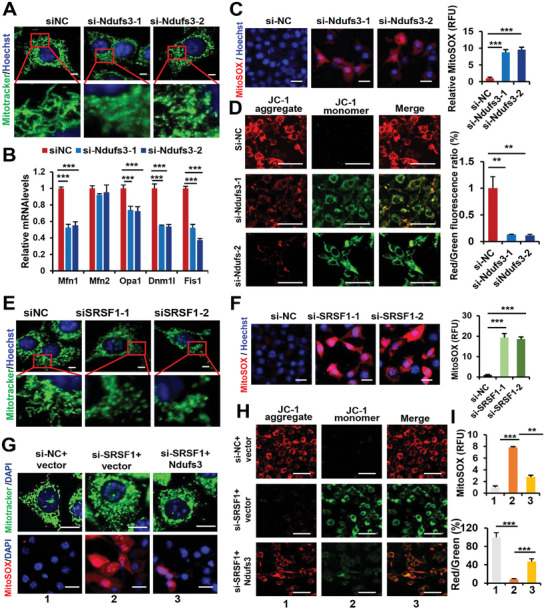
SRSF1 regulates mitochondrial homeostasis through controlling the inclusion of Ndufs3 exon 6 in brown adipocytes. A) Primary brown adipocytes were transfected with either Ndufs3‐specific siRNAs (siNdufs3‐1, siNdufs3‐2) or control siRNA (siNC) for 48 h. Representative images of cells stained with Mitotracker green are displayed. Enlarged images reveal the presence of fragmented and degenerated mitochondria. Nuclei were stained with Hoechst. Scale bar, 5 µm. B) q‐PCR analysis was performed to assess the expression of genes involved in the regulation of mitochondrial dynamics in cells described in (A). C) mtROS detection was carried out using MitoSOX staining (red) in cells described in (A). Scale bar, 20 µm. The quantification of relative fluorescence units (RFU) was shown in the right graph. D) Mitochondrial membrane potential was assessed using JC‐1 staining in cells described in (A). Green fluorescence indicated low membrane potential, while red fluorescence indicated high membrane potential and accumulation of JC‐1 in mitochondria. Scale bar, 50 µm. The ratio of red/green fluorescence was quantified and shown in the right graph. E) Brown adipocytes were transfected with SRSF1‐specific siRNAs (siSRSF1‐1, siSRSF1‐2) or siNC for 48 h. Representative images of cells stained with Mitotracker green are displayed. Enlarged images reveal the presence of fragmented and degenerated mitochondria. F) mtROS detection was carried out using MitoSOX staining (red) in cells described in (E). Scale bar, 20 µm. The quantification of relative fluorescence units (RFU) was shown in the right graph. G) Brown adipocytes were co‐transfected with siNC and vector plasmid, siSRSF1 and vector plasmid, or siSRSF1 and Ndufs3 plasmid for 48 h. Representative images of Mitotracker staining (green, top panel) and MitoSOX staining (red, bottom panel) were shown. Scale bars: 10 µm (top panel), 20 µm (bottom panel). H) Mitochondrial membrane potential was detected using JC‐1 staining in the cells described in panel (G). I) Quantification of RFU for the experiment described in (G) was illustrated on the top graph. Quantification of the red/green fluorescence ratio for the experiment described in (H) was presented on the bottom graph. ^*^ *p* < 0.05, ^**^ *p* < 0.01, ^***^ *p*,0.001. Data represent the mean ± SEM.

In comparison to cells treated with siNC, brown adipocytes treated with siNdufs3 displayed markedly fragmented mitochondria. These mitochondria did not exhibit the typical elongated rod shape but appeared swollen and spherical (**Figure** **9**A). Furthermore, the expression levels of genes associated with mitochondrial dynamics exhibited a significant decrease in Ndufs3‐knockdown cells (Figure 9B). Consistently, Ndufs3 knockdown resulted in an elevated production of mtROS, as evidenced by MitoSOX staining, a critical method used for assessing mitochondrial oxidative stress levels (Figure 9C). Using the cyanine dye JC‐1 to measure membrane potential, we further observed a significant reduction in membrane potential in adipocytes upon Ndufs3 knockdown (Figure 9D).

Similarly, the knockdown of SRSF1 in primary brown adipocytes also had detrimental effects on mitochondrial homeostasis. These effects included a significant increase in the presence of fragmented and degenerated mitochondria (Figure 9E), an elevation in mtROS production (Figure 9F), and perturbation of mitochondrial membrane potential (Figure S8B,C). Notably, these observations appeared to be more pronounced compared the effects observed in primary adipocytes upon Ndufs3 knockdown, emphasizing the critical roles of both SRSF1 and Ndufs3 in maintaining mitochondrial homeostasis and function.

To investigate deeper into the role of Ndufs3 as a mediator of SRSF1's influence on mitochondria within brown adipocyte, we conducted an experiment involving the introduction of Ndufs3 plasmids or empty‐vector controls into SRSF1‐knockdown cells (Figure S8D, Supporting Information). Intriguingly, our observations demonstrated that the expression of native Ndufs3 alleviates the adverse mitochondrial effects resulting from SRSF1 knockdown. These improvements included the reduction of fragmented mitochondria, the mitigation of increased mtROS production and the restoration of disrupted membrane potential (Figure 9G–I). Collectively, these findings underscored the crucial role of SRSF1‐regulated splicing control on Ndufs3 in maintaining mitochondria integrity and function in primary brown adipocytes.

### 2.11 SRSF1‐Regulated Splicing of Ndufs3 Influences Thermogenesis in Mature Brown Adipocytes

**Figure 10:**
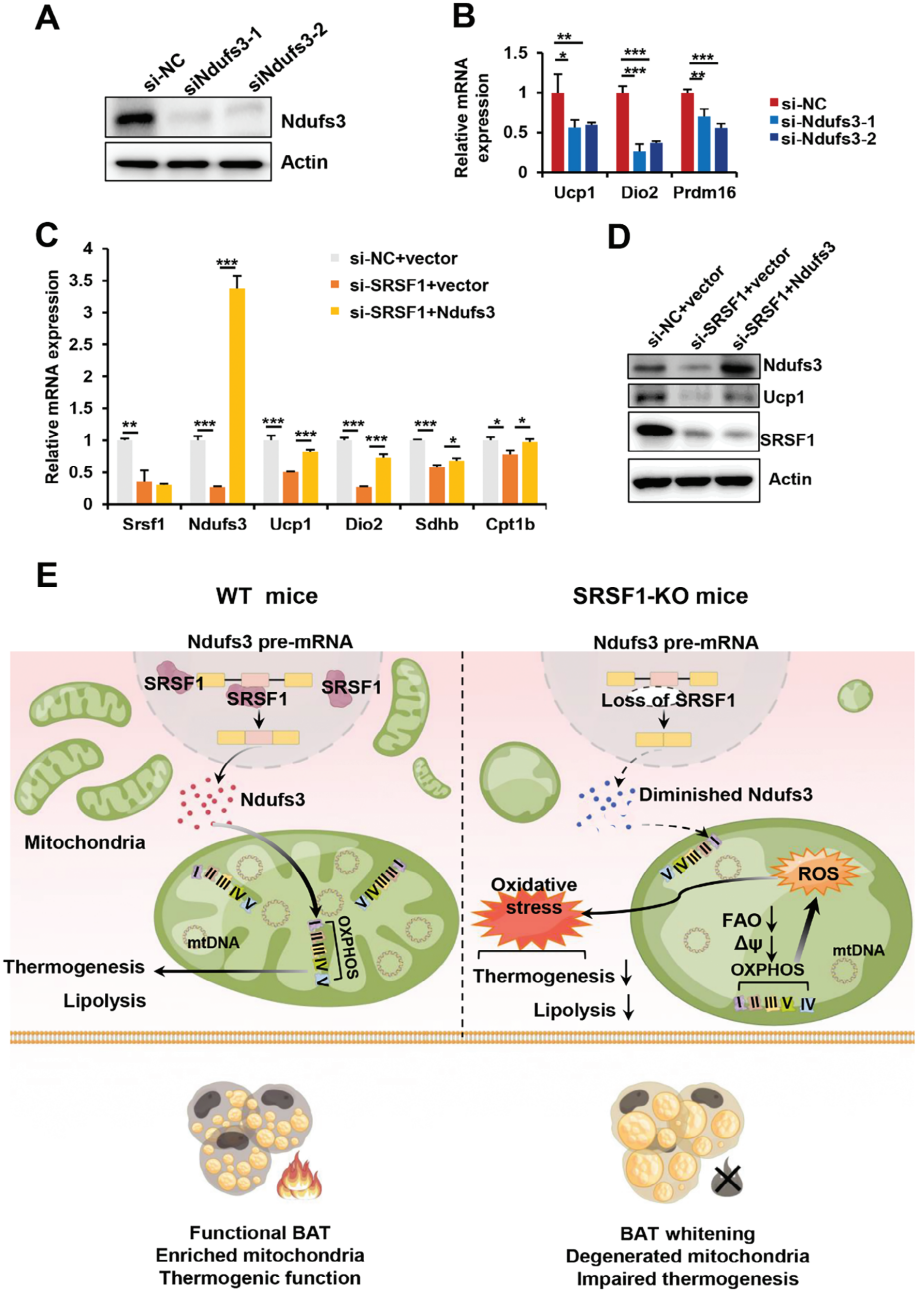
SRSF1‐regulated splicing of Ndufs3 plays a significant role in influencing thermogenesis in mature brown adipocytes. A,B) Primary brown adipocytes were transiently transfected with Ndufs3 siRNA or siNC, followed by induction of differentiation into mature adipocytes for the following experiments. Ndufs3 knockdown efficiency was confirmed by western blot (A). The expression of thermogenic genes was then assessed using q‐PCR in these cells (B). C) Brown adipocytes were co‐transfected with siNC and vector plasmid, siSRSF1 and vector plasmid, or siSRSF1 and Ndufs3 plasmid respectively, followed by induction of differentiation into mature adipocytes. The expression levels of genes involved in thermogenesis, mitochondrial OXPHOS, and FAO were measured by q‐PCR. The expression level of each gene was normalized to β‐Actin as an internal control (n = 3). D) Western blot analysis was conducted to assess the protein levels of Srsf1, Ndufs3, and Uc1 in the mature brown adipocytes described in panel (C). E) A functional model for SRSF1's role in the regulation of Ndufs3 splicing and its control over thermogenesis in BAT. Our model demonstrates that SRSF1 effectively binds to exon 6 of Ndufs3 pre‐mRNA, thereby promoting its inclusion in the final mRNA transcript. Conversely, the deficiency of SRSF1 results in impaired splicing of Ndufs3 and subsequently reduced levels of Ndufs3 proteins. Consequently, this deficiency triggers an increase in mtROS generation and disrupts mitochondrial function within brown adipocytes, ultimately compromising the thermogenic capacity of BAT. ^*^ *p* < 0.05, ^**^ *p* < 0.01, ^***^p,0.001. Data represent the mean ± SEM.

We next aimed to investigate the role of SRSF1‐regulated splicing of Ndufs3 in the regulation of thermogenesis in mature brown adipocytes. To investigate this, we conducted several experiments. First, we transiently transfected brown adipocytes with either Ndufs3 siRNA or siNC and allowed them to differentiate into mature adipocytes. As shown in **Figure** **10**A,B, both si‐Ndufs3‐1 and si‐Ndufs3‐2 significantly reduced the expression of thermogenic genes such as Ucp1, Dio2 and Prdm16 after brown adipocyte differentiation compared to cells treated with si‐NC.

As expected, SRSF1 knockdown affected the expression of genes such as Ndufs3, Ucp1, Dio2, Shdb and Cpt1b in mature brown adipocytes. Interestingly, overexpression of Ndufs3 in SRSF1‐knockdown cells significantly restored the expression of these genes (Figure 10C). In addition, western blot analysis further demonstrated that knockdown of SRSF1 resulted in significantly reduced levels of Ndufs3 and Ucp1, whereas the overexpression of Ndufs3 in SRSF1‐knockdowon cells led to a notable increase in the protein levels of Ucp1 without affecting SRSF1 levels (Figure 10D). These findings strong demonstrated that SRSF1‐regulated splicing of Ndufs3 influences thermogenesis in mature brown adipocytes.

## 3 Discussion

Emerging evidence underscores the significance of mutations or altered expression patterns in splicing regulators, along with aberrant splicing events, as common factors in diet‐induced obesity and metabolic dysregulation.^[^ ^23^ ^]^ Among these regulators, SRSF1 assumes a pivotal role as a splicing factor that governs AS in numerous genes crucial for essential cellular functions.^[^ ^24^ ^]^ However, its contribution to brown adipocyte thermogenesis and white adipocyte browning, two critical physiological processes tightly linked to energy expenditure, remains largely unexplored.

In this study, we introduce a transgenic mouse model, which allows us to investigate the specific effects of SRSF1 deficiency in mature adipocytes. Genetic ablation of SRSF1 results in the whitening of BAT and a reduction in thermogenic function, leading to metabolic disorders in mice. Furthermore, SRSF1 deficiency impedes the browning of WAT in response to cold challenge and β3‐agonist treatment. Importantly, our research reveals that SRSF1 deficiency disrupts mitochondrial homeostasis, leading to an excessive production of mtROS in BAT through the modification of the splicing of Ndufs3 (Figure 10E).

Mitochondria function is vital in maintaining the energy metabolism and thermogenic activity within BAT. Its malfunction leads to BAT whitening, characterized by the loss of its characteristic mitochondrial structure and function.^[^ ^25^ ^]^ Previous research has elucidated that deficiencies in APOO or Trib1 disrupt mitochondria structure and hinder oxidative phosphorylation in brown adipocytes, ultimately inducing BAT whitening.^[^ ^26^ ^]^ Similarly, our study unveiled noticeable BAT whitening and mitochondrial dysfunction in SRSF1 knockout mice. Through snRNA‐seq analysis, we observed that mature brown adipocyte subpopulations BA‐1 and BA‐2 predominantly occupied the BAT of WT mice, while they were replaced by white adipocyte subpopulation (WA) and mature brown adipocyte subpopulation BA‐3 within the BAT of KO mice. Although we noted increased adipocyte stem and progenitor populations in the BAT of KO mice, adipocyte differentiation appears similar between the two groups. Thus, BAT whitening in KO mice doesnot appear to result from abnormal adipocyte differentiation but rather emerges from mitochondria dysfunction, which possibly triggers the transformation of BA‐1 and BA‐2 subpopulations.

Previous research has investigated NOVA's role in governing the AS of JNK1 and JNK2 genes, integral component of the signaling pathway that regulates thermogenic gene expression.^[^ ^27^ ^]^ Additionally, another splicing factor, RBM4a, has been shown to oversees the AS of PRDM16, a pivotal transcription factor in brown adipogenesis, enhancing its impact on the development of brown adipocytes.^[^ ^28^ ^]^ In line with these previous findings, our research highlights the critical role of SRSF1 in regulating mitochondrial function and BAT thermogenesis in mice. Notably, our investigation reveals that the deficiency of SRSF1 results in alterations in the inclusion of alternative or constitutive exons in genes related to mitochondria function within BAT. We have provided substantial evidence that SRSF1 plays a critical role in promoting the inclusion of constitutive exon 6 of Ndufs3 pre‐mRNA. The skipping of exon 6 results in a decrease in the production of functional proteins, a critical subunit of mitochondrial complex I, leading to mitochondrial structural damage and dysfunction. Previous research emphasized SRSF1's role in adipocyte differentiation by influencing the AS of key genes such as Rps6kb1 and Pparγ.^[^ ^29^ ^]^ Yet, in Adipoq‐Cre‐mediated SRSF1 knockout WAT and BAT (Figure S9, Supporting Information), we did not observe abnormal isoforms for these two genes. This suggests that SRSF1 likely regulates different splicing events to maintain the physiological functions of mature adipocytes, differing from its role during adipocyte differentiation process.

D'Angelo et al. conducted an extensive investigation into the role of Ndufs3 and presented compelling evidence of how its deletion leads to a partial reduction in CI activity.^[^ ^11b^ ^]^ Even a modest decrease in CI activity can rapidly results in partial depolarization of the mitochondrial membrane, leading to uncontrolled accumulation of mtROS and alterations in mitochondrial morphology and dynamics. These changes invariably impact overall mitochondrial function.^[^ ^30^ ^]^ Similarly, we observed that direct knockdown of Ndufs3 or the skipping of exon 6 in Ndufs3 in the absence of SRSF1 led to a decrease in Ndufs3 proteins. This reduction disrupted mitochondrial homeostasis, resulting in increased mitochondrial fragmentation, excessive accumulation of mtROS, and mitochondrial membrane depolarization. Interestingly, SRSF1 knockdown resulted in more severe impairments in mitochondrial functions. Nevertheless, overexpressing Ndufs3 in SRSF1‐knockdown brown adipocytes effectively restored mitochondrial functions and increased the expression of thermogenic genes. Therefore, our findings underscore the vital significance of SRSF1‐regulated inclusion of Ndufs3 exon 6 in maintaining mitochondria homeostasis and BAT activity.

It is well‐established that WAT can undergo a phenomenon referred to as “beiging”, wherein it adopts brown adipocyte‐like characteristics in response to specific environmental or hormonal cues. This process plays a crucial role in thermogenesis, energy expenditure, and glucose homeostasis, making it as a promising target for addressing obesity and diabetes.^[^ ^31^ ^]^ Previous studies have suggested that mitochondrial dysfunction is linked to a phenotype marked by the suppression of WAT browning.^[^ ^26^, ^32^ ^]^ In eWAT of SRSF1‐deficient mice, we observed exon 6 skipping in Ndufs3 pre‐mRNA along with increased apoptosis‐related proteins, indicating potential impairment in WAT functions (Figure S10, Supporting Information). It is possible that mitochondria dysfunction plays a crucial role in the impaired browning of WAT observed in SRSF1‐deficient mice in response to cold exposure or agonist stimulation. However, further investigation is needed to uncover the precise mechanism by which SRSF1 deficiency affects the beiging process within WAT. Nevertheless, our findings provided valuable insights into the complex regulatory mechanisms governing beige adipocyte formation, highlighting the significant role for SRSF1 in this intricate process.

In conclusion, our study has unveiled a novel role for SRSF1 in the control of BAT thermogenesis. Our findings illustrate how SRSF1 influences the splicing of Ndufs3, a key player in mitochondria homeostasis and BAT function. These results contribute significantly to our understanding of the molecular intricacies governing adipose tissue metabolism and function, providing valuable insights into its physiological functions.

## 4 Experimental Section

### Animals

All experiments were conducted in accordance with the guidelines of the Institutional Animal Care and Use Committee of Shanghai Institute of Nutrition and Health, Chinese Academy of Sciences. SRSF1 ^flox/flox^ mice were described previously^[^ ^33^ ^]^ and provided by Dr. Fu XD (Westlake University). SRSF1^flox/flox^ mice were crossed with transgenic C57BL/6J mice expressing Cre recombinase driven by the adiponectin (Adipoq) promoter, which is active exclusively in adipose tissue. Littermates without the Cre gene (SRSF1^flox/flox^) were used as controls. Only males were used for further experiments, and littermates were compared in all studies. Body weight was measured weekly, food intake was calculated by measuring daily food consumption. For metabolic studies, mice were individually housed in metabolic cages (CLAMS‐16, Columbus Instruments), with free access to food and water. The oxygen consumption rate was monitored for 48 h. In the CL316, 243 treatment experiment, the mice were injected intraperitoneally with CL316, 243 at a dose of 1 mg·kg^−1^ at the 29.5 h mark during the 48 h period. The CL316243 continuous injection experiment involved daily intraperitoneal injection of CL316243 (1 mg kg^−1^) into mice daily for 7 days starting at 8 weeks of age, with a follow‐up experiment on the 8th day. For tissue collection, mice were sacrificed and perfused with ice‐cold PBS. Different adipose tissue depots (inguinal WAT, epididymal WAT, and interscapular BAT), and the liver were collected, weighed, and immediately frozen in liquid nitrogen.

### Blood Biochemical Analysis

Plasma triglyceride (TGs), total cholesterol (TC), high‐density lipoprotein (HDL‐C), and low‐density lipoprotein (LDL‐C) levels were enzymatically measured using an automatic analyzer (EnSpire Multimode Plate Reader, PerkinElmer, American). The reagents used for the analysis were purchased from Nanjing Jiancheng Bioengineering Institute, China. Blood glucose levels were measured using an Optium Xceed meter (Abbott Diabetes Care).

### Glucose and Insulin Tolerance Test

In the glucose tolerance test experiment, mice were fasted for 16 hours and then injected intraperitoneally with D‐glucose (1 g kg^−1^). In the insulin tolerance test experiment, mice were fasted for 8 h and then injected intraperitoneally with human insulin (Novolin) (0.75 U kg^−1^). Blood glucose levels were measured at indicated times using an Optium Xceed meter, from the tail vein.

### Histology and Immunohistochemistry

For H&E staining, iWAT, eWAT, BAT, and liver tissues were fixed in 10% neutral‐buffered formalin, then embedded in paraffin, and cut into 7 µm sections. HE staining was performed on these sections. Representative images were obtained from at least three independent experiments. To measure fat cell size, the H&E‐stained sections of at least three individual samples in each group were analyzed. The average diameter of adipocyte cells was determined using NIH Image J software from the National Institutes of Health. For immunohistochemistry, BAT, iWAT, and eWAT were fixed in 4% PFA overnight, dehydrated, and embedded in paraffin for sectioning. Antibodies for UCP1 (U6382, Sigma) were used for immunohistochemical staining. For immunofluorescence staining, the mice were anesthetized with isoflurane and perfused with phosphate buffered saline (PBS) through the aorta. Afterward, they were perfused with chilled 4% paraformaldehyde in phosphate buffer (pH 7.0). Brown adipose tissue was dissected, stored overnight at 4 °C in 25% sucrose, embedded in OCT compound, and cut at 15–35 µm using a cryostat. The tissue sections were then collected on slides. The slides were first washed in 0.1 m PBS and then blocked in a solution of 5% normal goat serum with 0.1% Triton X‐100, which was prepared in 0.1 m PBS, for a duration of 1 h. After that, the slides were incubated overnight at 4 °C with primary antibodies. The nuclei were stained with DAPI (1 µg mL^−1^). For BODIPY (D3922, Invitrogen) staining, 10 µm thick liver cryostat sections were used. The sections were rinsed with PBS and then stained with 1 µg mL^−1^ BODIPY at room temperature for 30 min. Immunofluorescence images were captured using a Zeiss LSM880NLO Confocal Microscope.

### Transmission Electron Microscopy Imaging

For transmission electron microscopy, BAT samples were fixed overnight at 4 °C with 2.5% glutaraldehyde (pH 7.4), and then postfixed at room temperature for 1 h with 2.0% osmium tetroxide. Thin sections were stained with uranyl acetate and lead citrate before being examined using a transmission electron microscope (Hitachi H‐7650, Japan). The representative images were obtained from three independent experiments.

### RNA‐Seq and Data Analysis

Total RNA isolated from KO and WT mice was used for paired‐end RNA‐Seq using an Illumina HiSeq 2000 system, following the manufacturer's instructions. Reads mapping and data analysis between the two samples were conducted as previously described. The raw sequence data have been submitted to the Gene Expression Omnibus under accession number PRJNA999070.

### Cold‐Stress Experiments

To induce short periods of cold stimulation, mice were individually placed in cages inside a freezer set at 4 °C without food. For longer durations of cold stimulation, mice were exposed to 4 °C for 24 h, during which they were adequately provided with food. The core body temperature was monitored using a rectal probe (TES‐1310, TES Electrical Electronic Corp).

### Infrared Thermography

An infrared camera (Magnity Electronics Co., Ltd, China) was utilized to measure interscapular surface temperature or entire body area or of mice. The thermal imaging was performed on all animals immediately after anesthesia. Multiple infrared photographs were taken of each animal during the light phase. The infrared analysis software (ThermoX) was employed to identify the maximum temperatures in the interscapular region from all photos of the same animal. A minimum of four photos were captured and analyzed for each animal/group.

### Protein Extraction and Western Blot Analysis

Tissue and cell lysates were prepared using RIPA buffer supplemented with a proteinase inhibitor cocktail (Roche), PMSF, and a phosphorylase inhibitor (Roche). Immunoblotting was performed using the following primary antibodies: SRSF1(MABE163, Merck, Kenilworth); AKT (4691; Cell Signaling Technology, Massachusetts); pAKT(S473) (9271, Cell Signaling Technology, Massachusetts); PGC‐1α (AF7736, Beyotime, Shanghai); UCP1 (U6382, Sigma, St Louis); ADRB3 (ab52623; Abcam); NDUFS3 (ab177471; Abcam); Caspase‐3 (AC030, Beyotime, Shanghai); BAX (A19684, Abclonal Technology, Wuhan); Bcl2 (68103‐1‐Ig, Proteintech, Wuhan); GAPDH (A19056, ABclonal, Wuhan); β‐Actin (sc‐47778; Santa Cruz Biotechnology, Dallas).

### RNA Isolation, RT‐PCR, qPCR, and Mitochondrial DNA Copy Number Quantification

Total RNAs were prepared using Trizol (Thermo Fisher Scientific, Massachusetts) following the manufacturer's instructions. Complementary DNA (cDNA) was synthesized by reverse transcription using oligo (dT) priming and M‐MLV Reverse Transcriptase (Promega Corporation) according to the manufacturer's protocol. The PCR products were analyzed by agarose gel electrophoresis and stained with ethidium bromide. Gene expression was measured using standard qPCR methods with the SYBR Premix Ex Taq kit (YEASEN Technology Company, Shanghai). Analysis was conducted on a QuantStudio Real‐Time PCR System (Applied Biosystems). The PCR primers used are listed in Table S1 (Supporting Information). To quantify the copy number of mitochondrial DNA, total DNA was extracted from brown adipose tissues using the Animal tissue DNA kit (3 101 050; Simgen, Hangzhou). To determine the copy number of mitochondrial DNA (mtDNA), a quantitative real‐time PCR‐based method was utilized, as described previously.^[^ ^34^ ^]^ The mtDNA content was quantified by calculating the ratio of the copy number of a mitochondrial gene (ND4) to that of a single‐copy nuclear gene (18S rRNA). The target mitochondrial DNA copy number was normalized to the nuclear gene 18s rRNA. The primers used for Real‐Time PCR are listed in Table S1 (Supporting Information).

### Isolation of Primary Brown Adipocytes and Adipocyte Differentiation

The stromal vascular fraction from interscapular BAT of male mice at postnatal day 1–2 was isolated.^[^ ^35^ ^]^ The tissues were minced in isolation buffer (Table S3, Supporting Information) and then filtered through a 70 µm cell strainer (352 350, Corning FALCON). The preadipocytes obtained were cultured in primary culture medium (Table S3, Supporting Information). Adipocyte differentiation was performed following a standard protocol. Two days after the preadipocytes reached confluence (day 0), differentiation was initiated by adding the induction medium (Table S3, Supporting Information). After 3 days, the medium was replaced with maintenance medium (Table S3, Supporting Information). The medium was changed daily until day 8.

### siRNAs and Plasmids

Different siRNA oligos were synthesized by Gene Pharma (Shanghai, China). Ndufs3 overexpression plasmids were purchased from Genescript (Nanjing, China). Minigenes were constructed by amplifying genomic sequences spanning exons 5–7 of Ndufs3 gene, which was then cloned into the PCDNA3.1 vector. Deletion or add‐in mutant derivatives were created based on the minigene plasmids. For transient transfection, siRNA or plasmids were transfected into primary adipocytes using either Lipofectamine RNAiMAX (Invitrogen, California) or Lipofectamine 2000 (Invitrogen, California), following the manufacturer's protocol. The sequences of the siRNA and plasmids can be found in Tables S1 and S2 (Supporting Information).

### Detection of Mitochondrial ROS Production in BAT

Detection of brown adipose tissue mtROS production was conducted following the previously described method.^[^ ^36^ ^]^ Briefly, freshly collected brown adipose tissue was immediately sectioned into 25 µm cryostat sections without fixation. The sections were then stained with 5 µm MitoSOX Red (YEASEN, Shanghai, China), a mitochondrial superoxide indicator, at 37 °C for 10 min. Subsequently, the nucleus was stained with DAPI for 10 min and the samples were visualized immediately using a Zeiss LSM880NLO Confocal Microscope. Quantification of fluorescence intensity was performed by examining five random fields per section and analyzed using ImageJ software.

### Mito Tracker Green, Mito SOX Red, and JC‐1 label in Primary Brown Adipocytes

To assess mitochondrial morphology and content in primary brown adipocytes, the cells with 100 nm green‐fluorescing MitoTracker Green (YEASEN Technology Company, Shanghai) were loaded for 30 min at 37 °C. Afterward, the cells were washed with warm complete culture medium. For measuring mitochondrial reactive oxygen species (ROS) levels, the cells with 5 µm MitoSOX Red were loaded for 10 min at 37 °C. Additionally, the nucleus was stained with Hoechest3342 for 10 min at 37 °C (ShareBio Technology Company, Shanghai). To examine mitochondrial membrane potential, the cells with 10 µg mL^−1^ JC‐1 (YEASEN Technology Company, Shanghai) were stained at 37 °C for 15 min in the dark. Subsequently, the cells were imaged using the confocal microscope immediately after washing them with dilution buffer. The excitation and emission wavelengths for each fluorescent dye were selected following the manufacturer's instructions. All data were obtained from experiments with a minimum of three replicates.

### RNA Immunoprecipitation

In exploring the interaction between SRSF1 protein and Ndufs3 pre‐mRNA, RNA immunoprecipitation (RIP) experiments in primary brown adipocytes were conducted using a specific antibody against SRSF1 (MABE163, Merck, Kenilworth). The experiments were performed following the guidelines provided by the manufacturer using the Magna RIP RNA‐Binding Protein Immunoprecipitation kit (Merck, Kenilworth). Cell lysates were prepared and incubated with beads‐antibody complexes. RNAs were extracted from the beads and reverse transcribed as mentioned earlier. The resulting cDNA products were then analyzed using gene‐specific primers for Ndufs3 through RT‐PCR or qPCR.

### Nuclei Isolation From Mouse Tissue and snRNA‐seq

The preparation of nucleus suspensions for experiments was conducted under constant ice‐cold conditions. Interscapular brown adipose tissue was harvested from 8‐week‐old control and adipose tissue‐specific SRSF1‐knockout mice. Fresh tissue samples were cut and lysed in 1 mL of nucleus lysis solution (NST) (Table S3, Supporting Information) on ice for 7 min. Once complete nucleus lysis was confirmed, 1 mL of ST Wash buffer (Table S3, Supporting Information) was added to resuspend the nuclei. The resuspension was then filtered through a 40 µm cell sieve, and the resulting filtrate was transferred to a 15 mL centrifuge tube. The cell sieve was washed with an appropriate amount of ST Wash buffer, and the wash buffer was combined with the nuclei filtrate. To ensure proper resuspension, the nuclei were resuspended. Finally, the nuclei were stained with Tissue Blue and counted through microscopic examination.

Nuclei were resuspended in PBS+1% BSA at a concentration of 700–1200/µL. The cDNA library amplification and DNA library construction were carried out using the 10x Genomics Chromium Next GEM Single Cell 3ʹ Reagent Kits v3.1 (1 000 268) and the Chromium Single Cell 3′/5′ Library Construction Kit (1 000 020), respectively, following the manufacturer's instructions. The libraries were sequenced on an Illumina Nova 6000 platform using PE150 mode.

### snRNA‐seq Data Processing

To process the data, the Cell Ranger software pipeline (version 3.1.0) provided by 10x Genomics was utilized. This pipeline facilitated several steps including demultiplexing of cellular barcodes, read mapping to the genome and transcriptome using the STAR aligner, and down‐sampling of reads to generate normalized aggregate data across samples. This resulted in a matrix of gene counts versus cells. BAT samples were collected separately from both WT and KO mice, resulting in two distinct samples. The reads obtained were aligned to the GRCm38 reference genome to generate unique molecular identifier (UMI) count matrices. The data from these sequenced samples were then aggregated using the aggr pipeline in Cell Ranger. Once the feature‐barcode matrix was obtained, consisting of 24341 nuclei, downstream analyses were performed using the R package Seurat. These analyses included quality control assessments, clustering of cells, cell annotation, and identification of differentially expressed genes (DEGs). The quality of the nuclei was evaluated based on metrics such as total UMI counts per nucleus, the number of detected genes per nucleus, and the proportion of mitochondrial gene counts. These quality control measures were crucial in ensuring the integrity and reliability of the subsequent analyses.

The identification of top variable genes across single cells followed the methodology outlined in Macosko et al.^[^ ^37^ ^]^ This involved calculating the average expression and dispersion for each gene, which were then categorized into multiple expression‐based bins. To reduce the dimensionality of the log‐transformed gene‐barcode matrices of the top variable genes, principal component analysis (PCA) was performed. Subsequently, cells were clustered using a graph‐based clustering approach, and the results were visualized in a 2D space using t‐distributed stochastic neighbor embedding (tSNE). To identify significantly differentially expressed genes between clusters, a likelihood ratio test was employed. This test simultaneously assessed changes in mean expression and the percentage of expressed cells. For unbiased cell type recognition of scRNA‐seq data and inference of the cell of origin for each single cell, the R package SingleR^[^ ^38^ ^]^ was utilized, a novel computational method. By applying SingleR, the cell types of each single cell were independently determined and identify their respective cell of origin. This approach allowed for unbiased cell type characterization and classification.

Differential expression analysis was conducted using the Seurat^[^ ^39^ ^]^ package to identify genes that exhibited significant changes. For a gene to be considered differentially expressed, it had to meet the following criteria: *p* value <0.05 and |log2foldchange| > 1 (or |log2foldchange| > 0.58). GO enrichment and KEGG pathway enrichment analysis of DEGs were performed using R and the hypergeometric distribution.

### GSVA Enrichment Analysis

To perform pathway enrichment analysis, the background gene set files from the KEGG database (https://www.kegg.jp/) was initially obtained. These files were downloaded and organized using the GSEABase package (v1.44.0). Next, the signal pathway activity values for individual cells using the GSVA package (v1.30.0) were calculated. This enabled us to assess the activity levels of various signaling pathways within each cell. To compare the differences in signal pathway activity among different groups, the LIMMA software package (v3.38.3) was employed. This package facilitated the statistical analysis and calculation of differential pathway activity. By comparing the activity levels of specific pathways between different groups, significant differences in pathway activation were successfully identified.

### Cell Development Trajectory

To infer the trajectory of cell differentiation from the Seurat object, the Monocle2 (v2.9.0) package was utilized. This package enables to conduct pseudotime analysis on the cells. Initially, the Seurat object to a CellDataSet object using the importCDS function of the Monocle2 package was converted. Subsequently, the ordering genes (qval < 0.01) were identified that exhibited differential expression among cell clusters. This was achieved by employing the differential GeneTest function of the Monocle2 package, which utilizes a negative binomial generalized linear model to assess gene expression changes. The reduceDimension function of the Monocle2 package was then employed to reduce the dimensionality of the CellDataSet object. This function employs unsupervised learning algorithms, such as independent component analysis or non‐negative matrix factorization, to identify latent factors that capture the primary sources of variation in the data. Finally, the differentiation trajectory of the cells was inferred using the orderCells function of the Monocle2 package. This function utilizes a minimum spanning tree algorithm to connect cells based on their similarity in the reduced dimensional space. Additionally, it assigns a pseudotime value to each cell based on its position along the trajectory.

### Data Availability

The snRNA‐seq data sets have been deposited in the GEO database under the accession number GSE238009.

### Statistical Analyses

Statistical analysis was performed using GraphPad Prism 8.0 software. The histograms display data from at least three independent experiments and were presented as the mean ± SEM. The sample size (n) for each statistical analysis is specified in the figure legend. Statistical significance between groups was determined using the two‐tailed unpaired Student's t‐test. Differences between values were considered statistically significant when ^*^ *p* < 0.05, ^**^p < 0.01, and ^***^ *p* < 0.001.

## Conflict of Interest

The authors declare no conflict of interest.

## Author Contributions

N.Y. and L.S. contributed equally to this work and are co‐first authors. Conception and design were performed by N.Y. and Y.F. Data acquisition and analysis were performed by N.Y., L.S., Q.P., R.S., Z.W., Z.X., X.Y., and Y.F.. Drafting of the manuscript was performed by N.Y. and Y.F..

## Supporting information

Supporting Information

## Data Availability

Research data are not shared.
