# Supplementary material for: SRSF1 Is Required for Mitochondrial Homeostasis and Thermogenic Function in Brown Adipocytes Through its Control of Ndufs3 Splicing
Source: Adv Sci (Weinh). 2024 Apr 3;11(21):2306871. doi: 10.1002/advs.202306871 (PMC11151030; doi:10.1002/advs.202306871)
Supplement: Supplementary file 1 — Supporting Information [file ADVS-11-2306871-s001.pdf]

ADVANCED SCIENCE
Open Access

Supporting Information

for *Adv. Sci.*, DOI 10.1002/advs.202306871

SRSF1 Is Required for Mitochondrial Homeostasis and Thermogenic Function in Brown Adipocytes Through its Control of Ndufs3 Splicing

Ningyang Yuan, Lei Shen, Qian Peng, Rula Sha, Zhenzhen Wang, Zhiqi Xie, Xue You and Ying Feng**

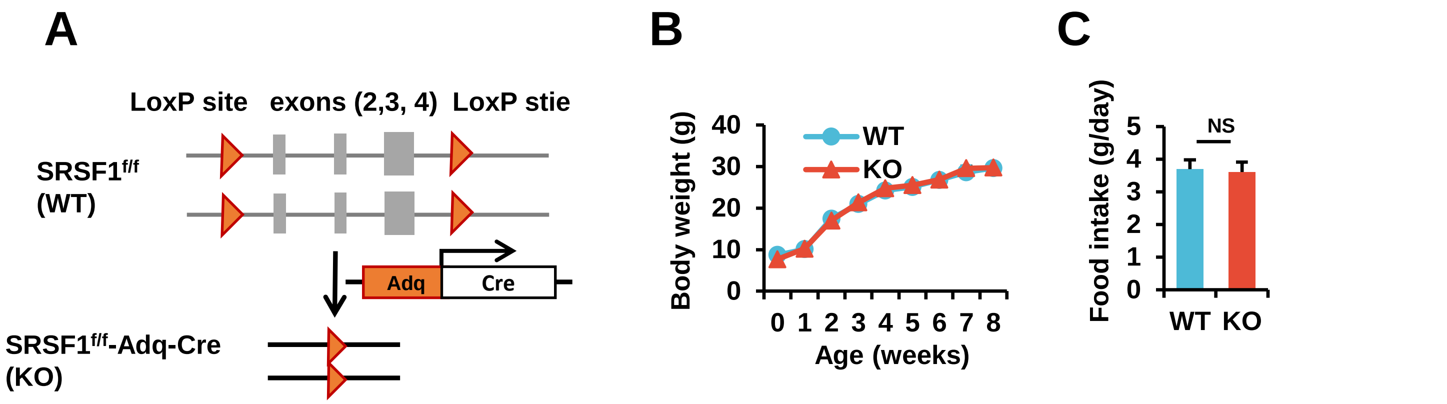

Figure S1. Body weights and food intake were compared between SRSF1 knockout mice and control mice. (A) Schematic diagrams illustrating the strategy for generating adipose tissue-specific SRSF1 knockout mice. (B) The growth curve of both WT and KO mice were presented fed a chow diet (n=8). (B) Quantification of average daily food intake in 8-week-old mice on a normal chow diet (n=8/group).

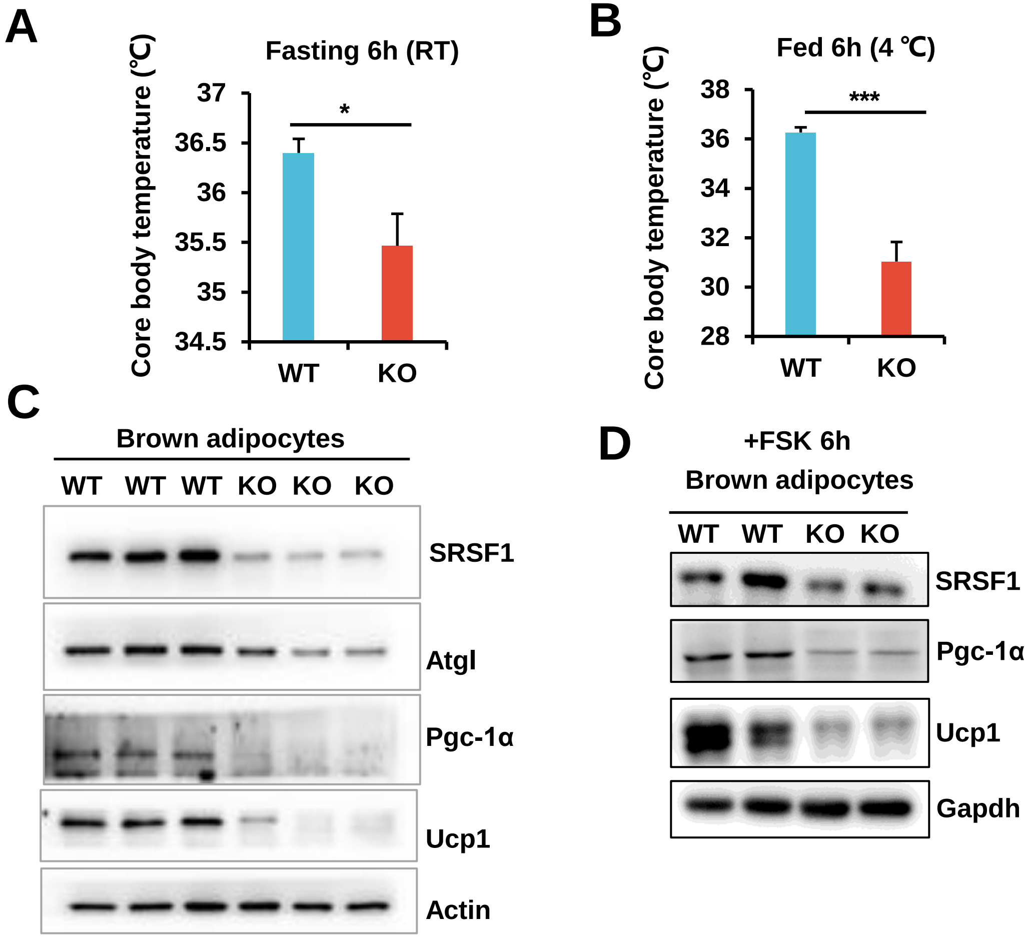

Figure S2. Impaired thermogenesis observed in KO mice stemmed from dysfunction in brown adipocytes. (A) Rectal temperatures of 8-week-old WT and KO mice were measured at room temperature (RT) after a 6-hour fasting (n=4). (B) Rectal temperatures of 8-week-old mice, individually housed, were measured after exposure to cold (4℃) (n=6) while generously provided with food during this period. (C) Primary brown adipocytes isolated from BAT of WT and KO mice at 1 day of birth were cultured in differentiation medium until day 8. Subsequently, proteins were extracted from the cells and analyzed by Western blotting to assess the expression of thermogenesis-related proteins, such as Ucp1, Pgc-1α, and Atgl. (D) Primary brown adipocytes were differentiated for 8 days and then treated with 10 μM FSK for 6 h. Western blot analysis was performed to measure SRSF1, Ucp1, and Pgc-1α protein.* $p<0.05$, ** $p<0.01$, *** $p<0.001$. Data represent the mean ± SEM.

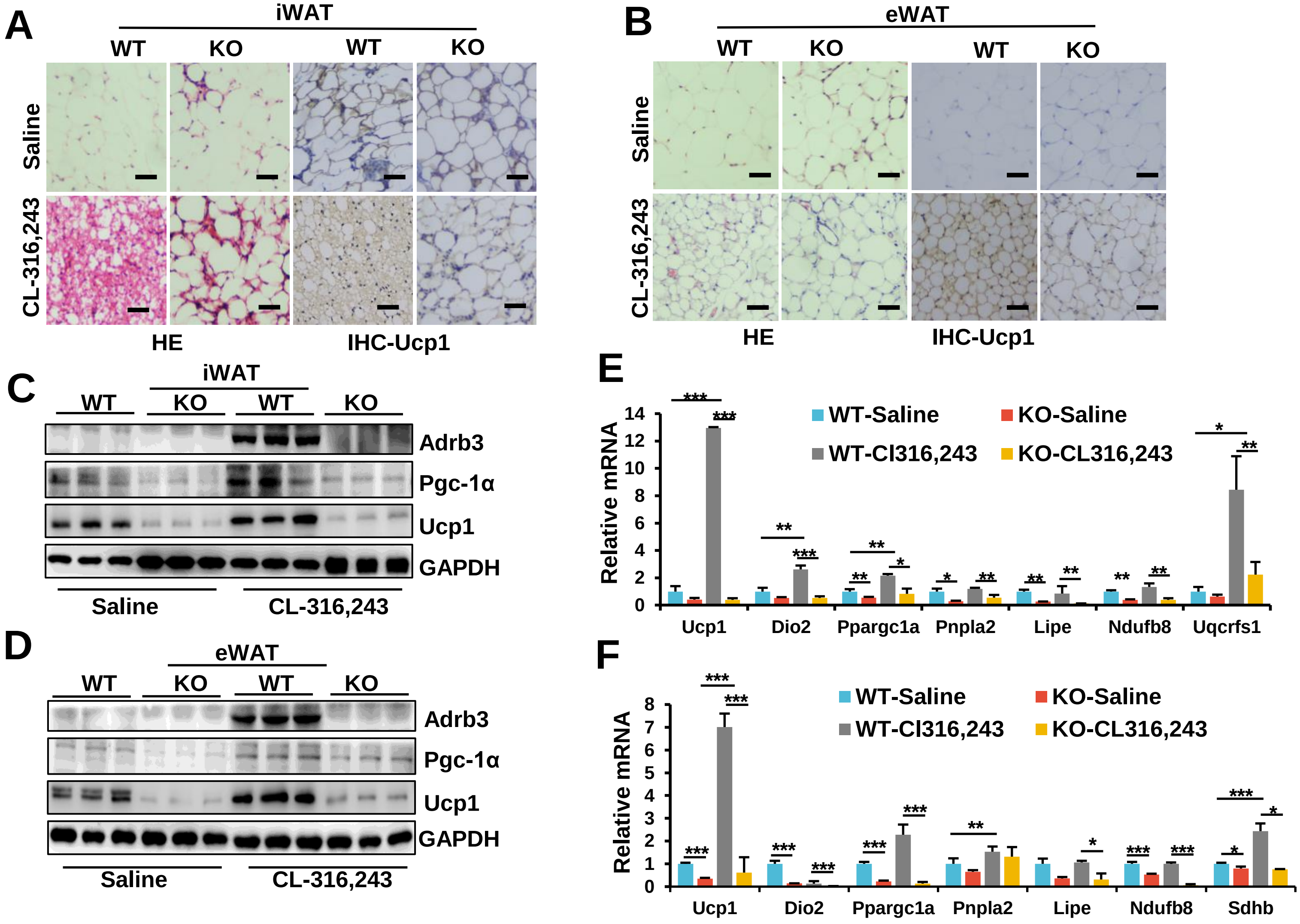

Figure S3. SRSF1 Regulates the browning of WAT following treatment with β3-adrenergic agonist. (A-B) Representative images of HE staining (left) and immunostaining of Ucp1 protein (right) in iWAT (A) and eWAT (B) from WT and KO mice following daily injections of CL-316,243 or saline for 7 days. Scale bar, 50μm. (C-D). Western blot detection of thermogenic proteins in iWAT (C) and eWAT (D) from WT and KO mice injected with CL-316,243 or saline for 7 days. (E-F) q-PCR detection of mRNA expression of thermogenic genes in iWAT (E) and eWAT (F) after CL-316,243 or saline treatment. * $p<0.05$, ** $p<0.01$, *** $p<0.001$. Data represent the mean ± SEM.

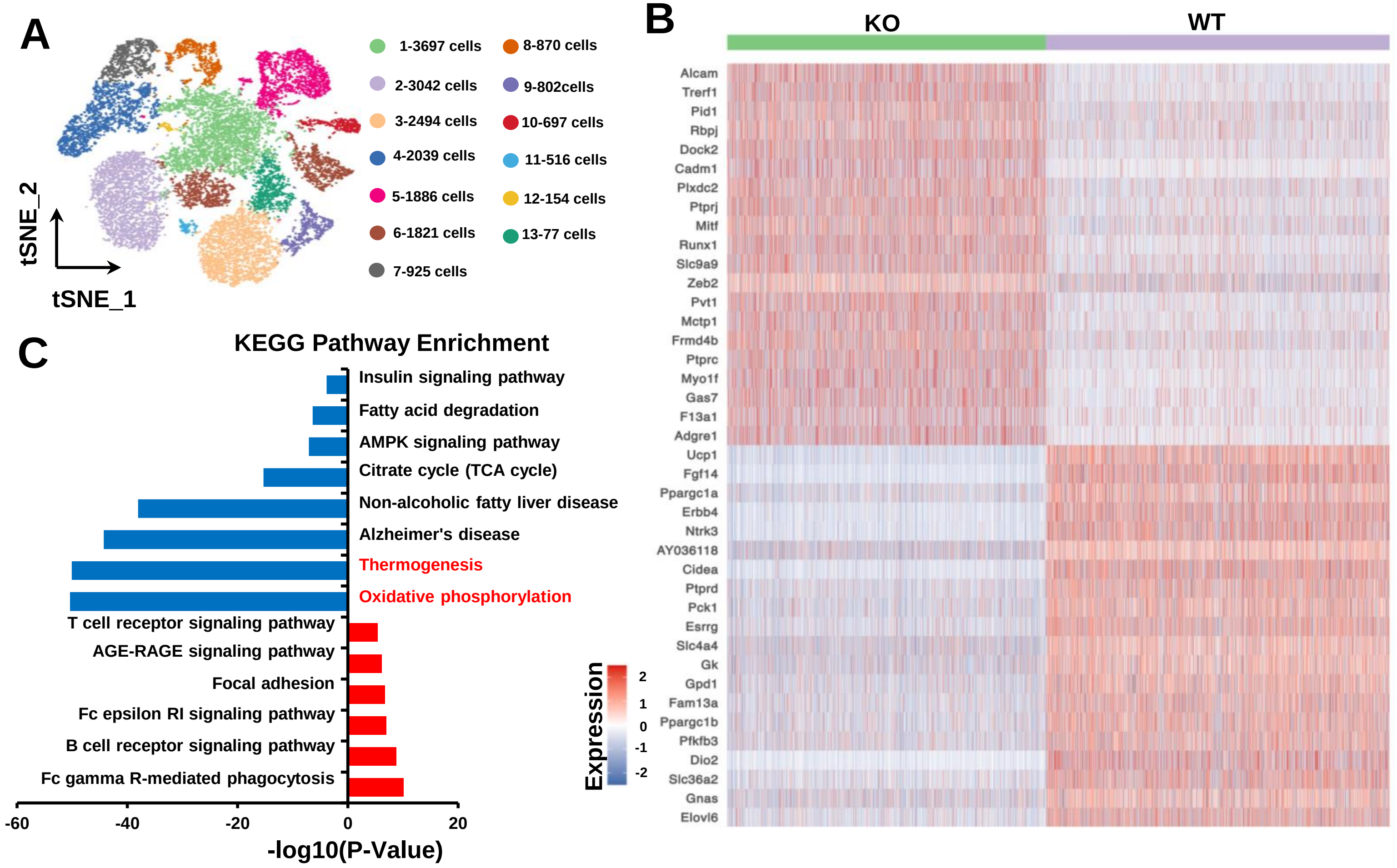

Figure S4. Analysis of differentially expressed genes and their pathway analysis between the two groups based on snRNA-seq. (A) t-SNE plot of the entire dataset (n = 19,020 nuclei). Cells were colored and labelled by clusters. (B) Heatmap of DEGs between the two groups. Top 20 DEGs and their scaled expression were shown. (C) KEGG pathway enrichment analysis was performed using Fisher exact test. Only KEGG pathways with a p-value less than $p < 0.05$ were considered significant and presented. The fold-enrichment of each pathway was indicated by a bar, with red bars representing upregulated pathways and blue bars representing downregulated pathways.

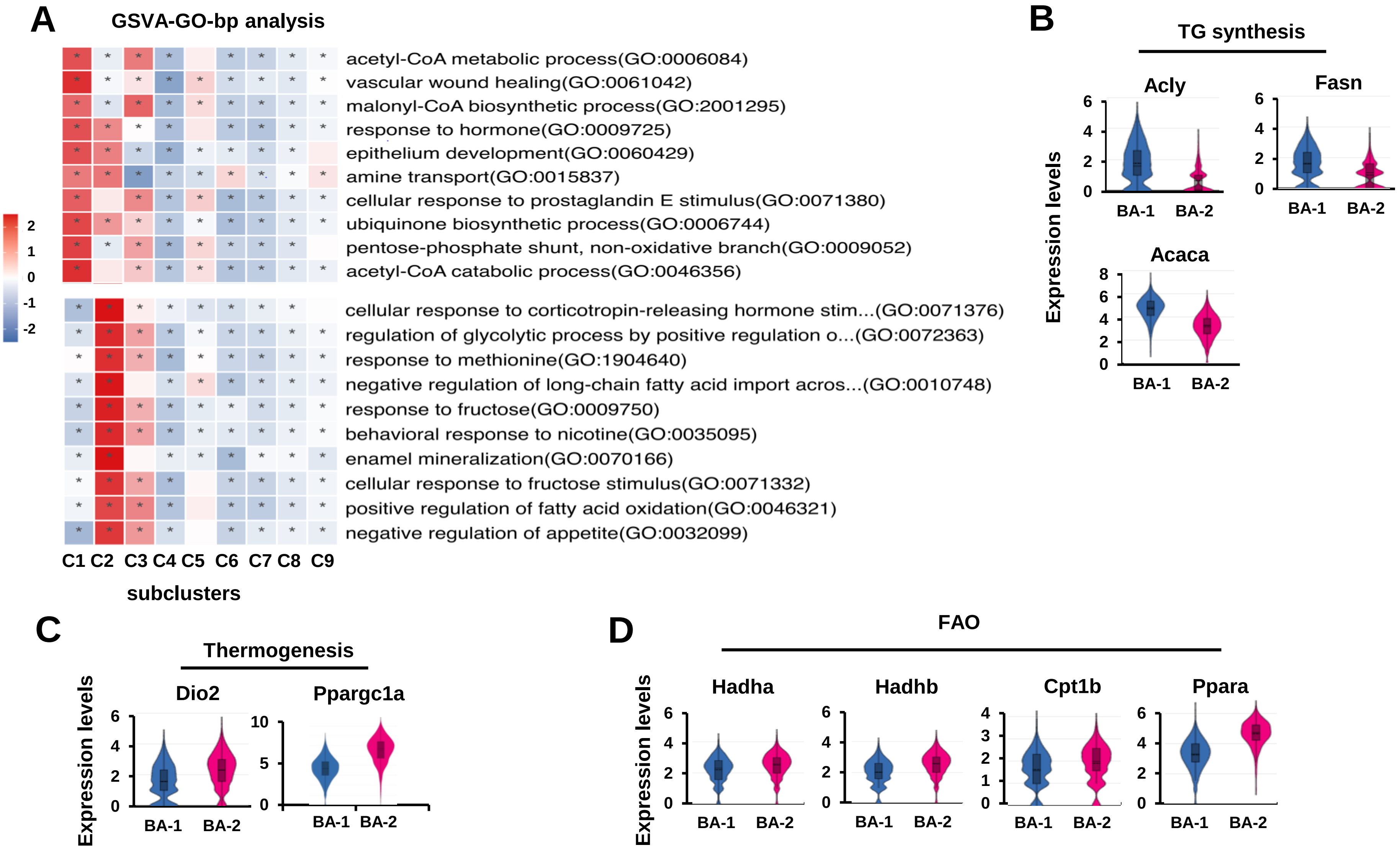

Figure S5. Two distinct types of brown adipocytes with different functions were identified in the BAT of WT mice. (A) GSVA was employed to examine the GO enrichment of biological processes within the C1 (BA-1) and C2 (BA-2). (B-D). Violin plots were used to visualize the expression levels of genes related to lipid synthesis (B), thermogenesis (C), and FAO(C).

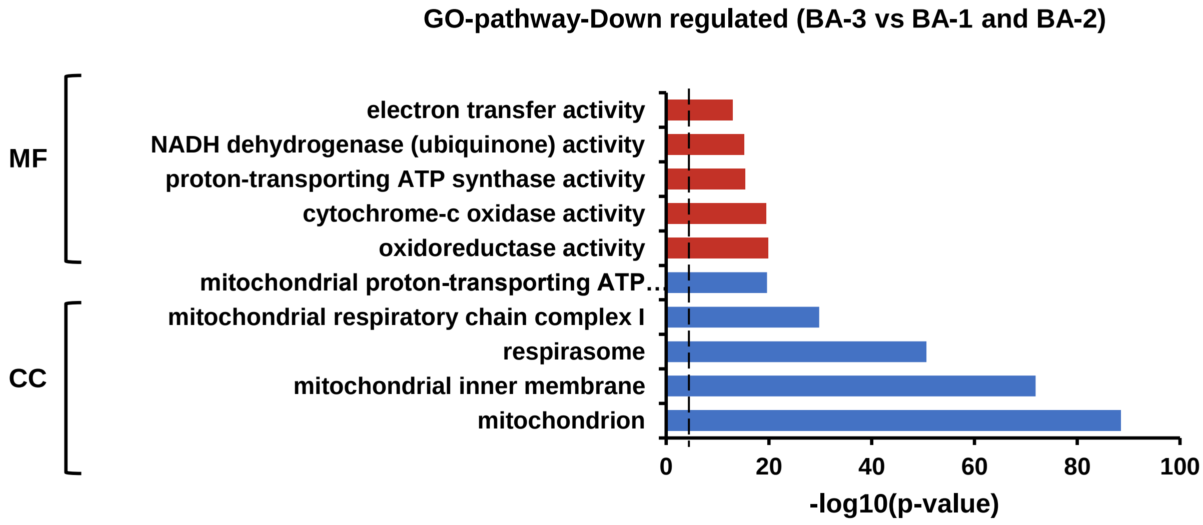

Figure S6. Significant enriched GO terms of downregulated genes in BA-3 compared to BA-1 and BA-2. Red, molecular function (MF); blue, cellular component (CC). The top 5 significant terms of each category in downregulated DEGs were displayed.

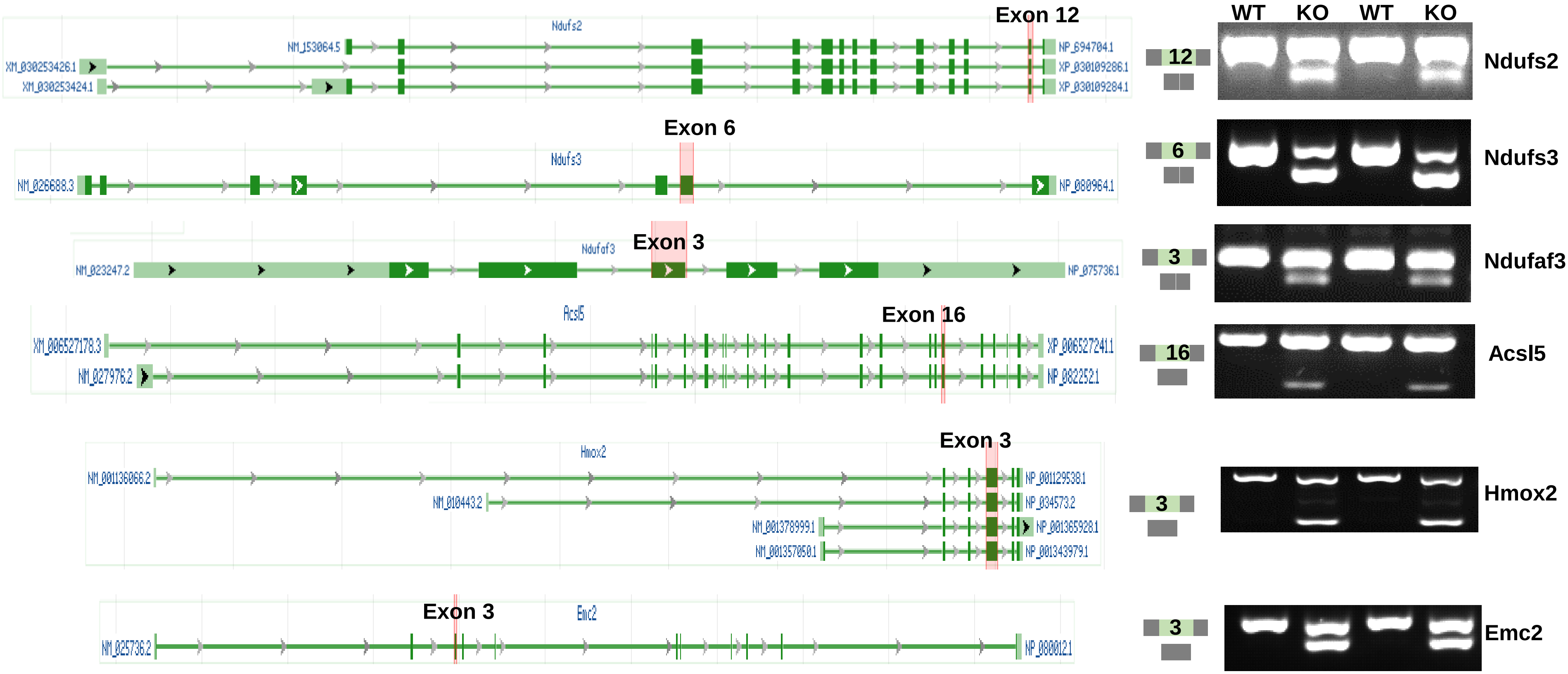

Figure S7. SRSF1 regulates the splicing of constitutive exons within BAT. The gene information is screenshotted from the NCBI website and the exon regulated by SRSF1 in each gene is marked (left). RT-PCR results indicted that the absence of SRSF1 leads to the skipping of these constitutive exons in BAT of KO mice compared to WT mice (right).

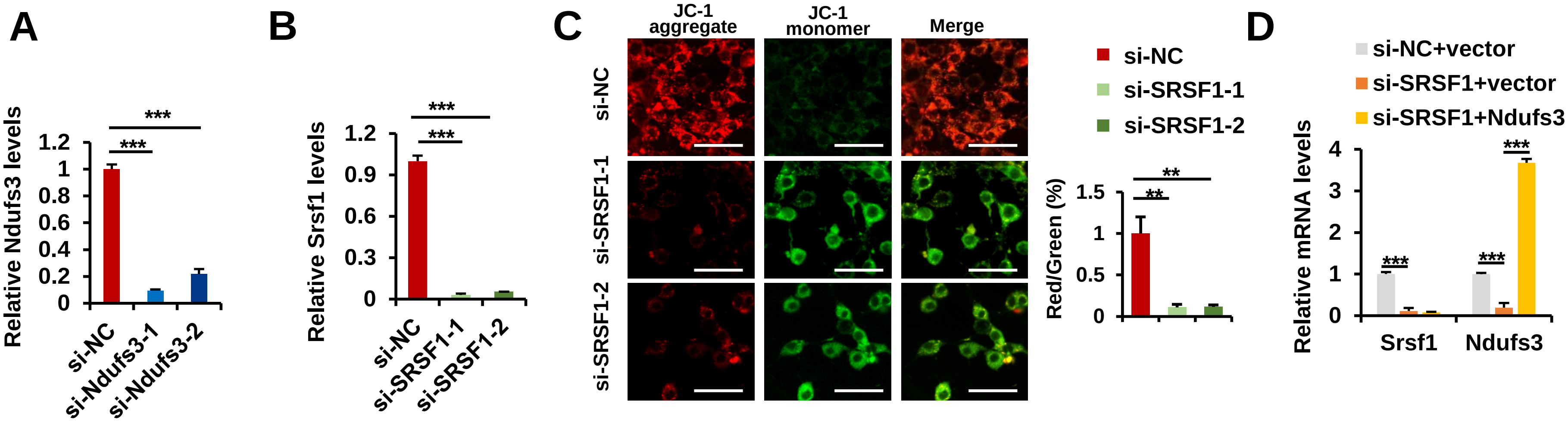

Figure S8. SRSF1 preserves mitochondrial function by controlling the inclusion of exon 6 in Ndufs3 in primary brown adipocytes. (A) Brown adipocytes were transiently transfected with Ndufs3 siRNA (siNdufs3-1, siNdufs3-2) or siNC for 48 hours. Knockdown efficiency of Ndufs3 was confirmed by qPCR. (B) Brown adipocytes were transiently transfected with SRSF1 siRNA (siSRSF1-1, siSRSF1-2) or siNC for 48 hours. Knockdown efficiency of SRSF1 was confirmed by qPCR. (C) Mitochondrial membrane potential was assessed using JC-1 staining in cells described in (B). Green fluorescence indicated low membrane potential, while red fluorescence indicated high membrane potential and accumulation of JC-1 in mitochondria. Scale bar, 50 μm. The ratio of red/green fluorescence was quantified and shown in the right graph. (D) Brown adipocytes were co-transfected with siNC and vector plasmid, siSRSF1 and vector plasmid, or siSRSF1 and Ndufs3 plasmid for 48 hours. The expression of SRSF1 and Ndufs3 in these cells was assessed by qPCR.

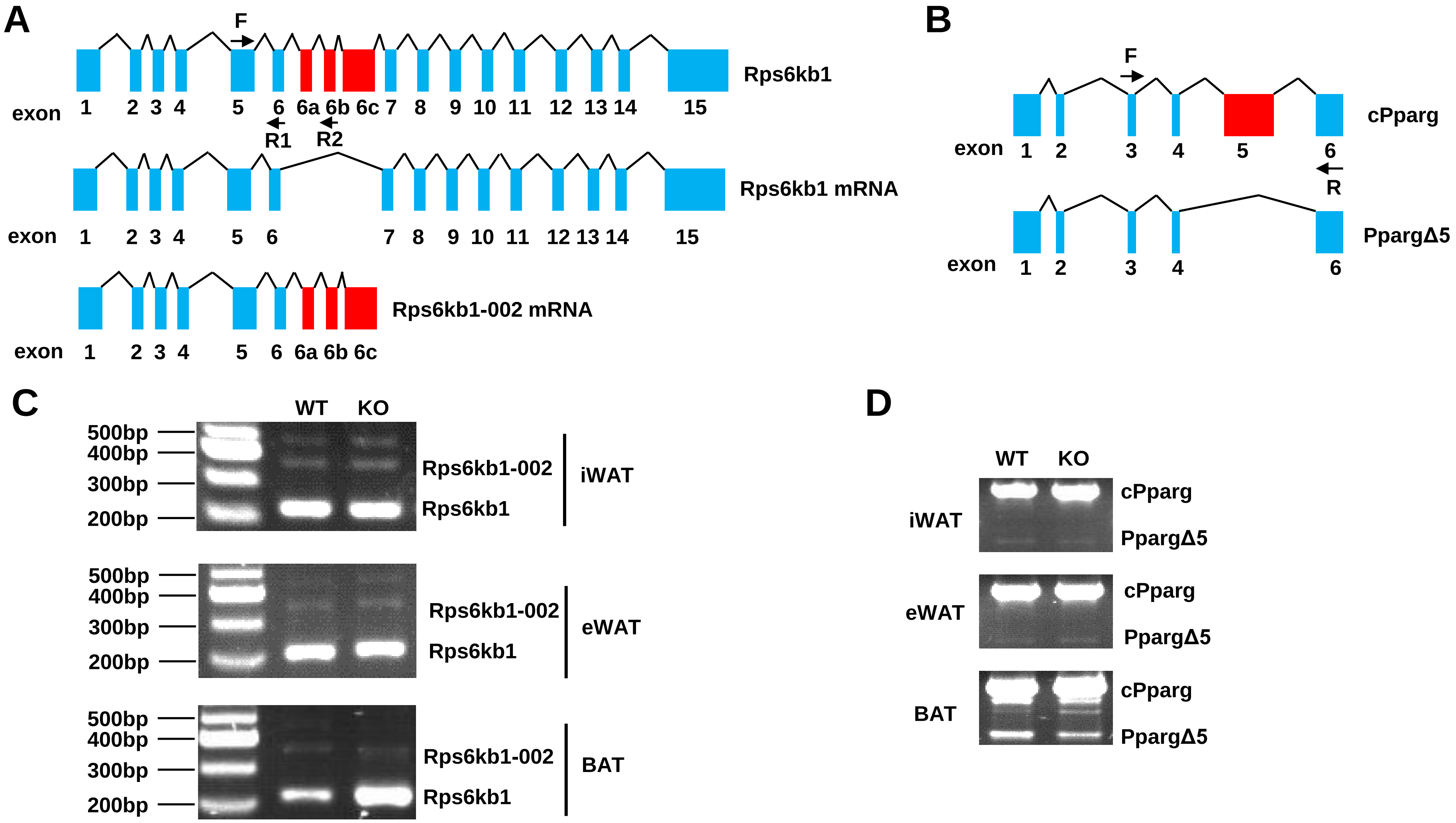

Figure S9. Splicing isoforms of Rps6kb1 and Pparg in WT and KO mice were examined. (A, B) Schematic representation illustrating the exon structures for the mouse Rps6kb1 and Pparg genes. Constitutive exons are indicated by blue boxes, while alternatively spliced exons are denoted by red boxes. (C and D) Detection of splicing patterns for Rps6kb1 (C) and Pparg (D) via RT-PCR in WAT and BAT of WT and KO mice.

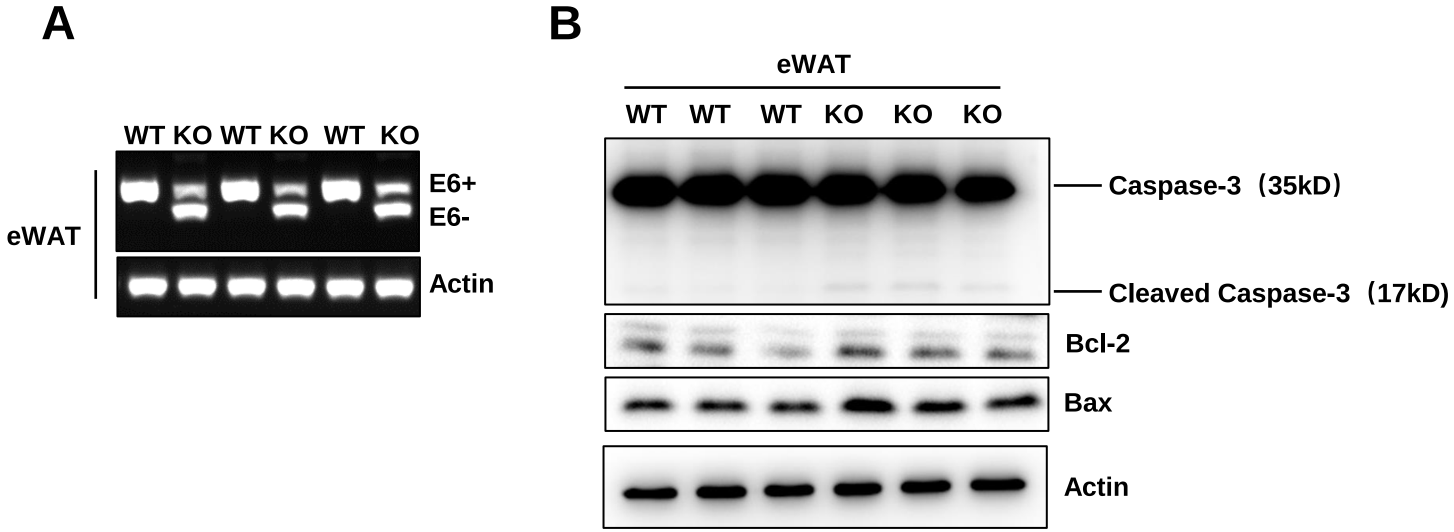

Figure S10. eWAT of KO mice exhibited Ndufs3 splicing defects and elevated apoptosis-related proteins. (A) RT-PCR analysis revealed alterations in the splicing isoforms of Ndufs3 in eWAT of KO mice. (B) Western blot analysis indicated higher levels of Bax, Bcl-2, and cleaved Caspase-3 in eWAT of KO mice compared to WT mice.

Table S1. Primers and siRNA sequences

**Sequences of q-PCR primers**		
**Gene**	**F(5'-3')**	**R(5'-3')**
Srsf1	GGCTACGACTACGACGGCTA	AGAGACAACCACTCTGTTCTCG
Cidea	CAGTCTGCAAGCAACCAAAG	GCCTTGAAGCTTGTGCATC
Dio2	CCACCTGACCACCTTTCACT	TGGTTCCGGTGCTTCTTAAC
Prdm16	CAGCCATAGAAACCATGACG	CTCTCCTTCCCCTTCCTGAG
Cox8b	TGTGGGGATCTCAGCCATAGT	AGTGGGCTAAGACCCATCCTG
Ucp1	CTGCCTCTCTCGGAAACAAG	TTTGGTTGGTTTTATTCGTGGTC
Ppargc1a	CCGAGAATTCATGGAGCAAT	TGTCTCTGCTGCTGTTCCTG
Aco2	AAGGGAAGTGCACCACAGAC	AGGTACAGGGCCAAACTCCT
Ndufb8	ACTGGGACCTAGACATGTACATCAG	CAGGCTGGTAGGAAGGGAAC
Lipe	TTCGCCATAGACCCAGAGTT	CGACAGCACCTCAATCTCAG
Mgll	GGGCAGAGTGAGGGAGAGAG	GACGTCGGGGTAGTCCTTCT
Pnpla2	TGAAGCAGGTGCCAACATTA	AACACGAGTCAGGGAGATGC
Uqcrfs1	CGTCCTATCGGCCACTTC	TCGCTTCACATCCAGAACAG
Sdhb	CCTATCGCTGGATGATCGAC	GCAGGTCTGTGTGCAGTTCA
F4/80	TGACTCACCTTGTGGTCCTAA	CTTCCCAGAATCCAGTCTTTCC
Cd11b	GGGAGGACAAAAACTGCCTCA	ACAACTAGGATCTTCGCAGCAT
CD68	TGTCTGATCTTGCTAGGACCG	GAGAGTAACGGCCTTTTTGTGA
Mgl1	TGAGAAAGGCTTTAAGAACTGGG	GACCACCTGTAGTGATGTGGG
Mrc1	CTCTGTTCAGCTATTGGACGC	CGGAATTTCTGGGATTCAGCTTC
Tnf-α	GACGTGGAACTGGCAGAAGAG	GCCACAAGCAGGAATGAGAAG
Ndufs3	TGGGACATGTTTGGAGTTTTC	AGCCAGTGAGGGGAAAGTCT
Ndufs3- T	TGAGAGGGAGCTTCGTTACG	GCTGGAGACAAGAAGCCTGA
Mfn1	TGATCTCAATTGCCACAAGC	GGAACCCAGGAATCGATGTA
Mfn2	GCTCTGGGACAAAGTTCTGC	TCTGAGCCCTCTGTGAGGAG
Opa1	CAAGCTTCAAAGACCCCAAC	GGGGCTGGTAGCCATATTTA
Dnml1	CCGGTGGATGATAAAAGTGC	TGTACTTTGCGGTTCCTTCA
Fis1	TAAAGTATGTGCGAGGGCTGT	CTTTCTTCATGGCCTTATCAATC
Cpt1b	AGCCATCGAGAACTCGTACC	GGGGCTGGTCCTACACTTCT
Nox1	GGAATTGCAGATGAGGAAGC	CAGAGTGAAGGGATGCCACT
App	GTGAGCGACGCCCTTCTC	GGTGTGCCAGTGAAGATGG
Trib3	GGCTCTCGGCTCCTTTACAT	AGCCTCGGACTCTGGGATAC
Mreg	TTCCTCCTTTGGAGCGACT	CTATCGTCATCCGCCTCTGT
Mtarc1	CTGTGAGGACGACACCTTGA	ACTGGAGGAGTGGGTTTGTG
Sod2	CAAGCGTGACTTTGGGTCTT	GCGACCTTGCTCCTTATTGA
Txn2	CCCCTCAGTACAATGCTGGT	TCCATCCTGGACGTTAAAGG
Oxr1	TTCCCACCAGGAAAGTTCAC	CCTGCCCTTGTTGATCCTTA
Actin	GGCTGTATTCCCCTCCATCG	CCAGTTGGTAACAATGCCATGT
**Sequences of genotyping RT-PCR primers**		
**Gene**	**F(5'-3')**	**R(5'-3')**
Cre	GCCTGCATTACCGGTCGATGC	CAGGGTGTTATAAGCAATCCCC

GT-SRSF1	ACTAATGTGGGAAGAATGGC	AAACTATTGCTCCCATCTGC
**Sequences of RT-PCR primers**		
Full length Ndufs3	ATGGCGGCGGCTGCAGCCAGG	TTACTTGGTTTCAGGCTTCT
**Sequence of splicing primers**		
**Gene**	**F(5'-3')**	**R(5'-3')**
Letmd1	GCGGACGTGAAGAACTTGAT	GTAGTTGGCAAAGGGTGGAA
Ndufs7	TCTGTACGGATCCTGGGTCT	TCCAGCTTGGTCACCACATA
Ndufaf3	GCCGTGTACATCGACAGCTA	CTGGACCTCCACAGCAATTC
Ndufas6	ACTGTGACAACCCACCACAC	CCAGCACCTCCAGTGTCAG
Ndufs2	GGGTATACTTGGTATCCGATGG	CGGTCTATTTCTCCGAACACA
Ndufs3	GATGAGCTGACACCCATTGA	GCTTCTTGTCTCCAGCTTCG
Emc2	TGAGAAAATGGCGAGAAGAAA	TTCAAATCGCATGCCTGTTA
Acsl5	CTCCAGTGGCTTGCAATTTT	TCAATCTTCTCTGGGGCAAT
Hmox2	TCAAAGACTTCTTGAAAGGAAACA	TCTGGCTGAGCAGCATAAAA
Nfs1	TGATCCTCGGGAGATCATTT	TTTCTGCAATTGGTTGCTTT
Cyp27a1	GGTCCAGGAACAGGTCAAGA	CTCGTTTAAGGCATCCGTGT
Atp5d	CTACGCTGACTGGAGCCTTT	AGTTCTGACTGCGCCTTCTC
Atp6ap1	ACCAGCGATATGCAGCTTTC	GTGGTCAGAGTGCTGATTGC
**Sequences of mtDNA q-PCR primers**		
**Gene**	**F(5'-3')**	**R(5'-3')**
18S rRNA	TAGAGGGACAAGTGGCGTTC	CGCTGAGCCAGTCAGTGT
ND4	TCGCCTATTCATCAGTAAGTCA	GGATTATGGTTCGGCTGTGTA
**siRNA sequences**		
**Gene**	**F(5'-3')**	**R(5'-3')**
siNdufs3-1	GAAGGAUCCUGACAGAUUATT	UAAUCUGUCAGGAUCCUUCTT
siNdufs3-2	UAAGAAGGAUCCUGACAGATT	UCUGUCAGGAUCCUUCUUATT
siSRSF1-1	GAGGCAGGUGAUGUAUGUUTT	AACAUACAUCACCUGCCUCTT
siSRSF1-2	CUGGCAGGACUUAAAGGAUTT	AUCCUUUAAGUCCUGCCAGTT
Ndufs3 (RNA RIP)	TGAGAGCCACTCTTTTCTAG	CACTGGCTATGTTGAGGTAG

Table S2. Plasmids

**Sequences of plasmids**
**Ndusf3:**<br>CTCGAGATGGCGGCGGCTGCAGCCAGGGTCTGGTGTCGTGGGCTCTTGGGGGCCGC<br>TTCCGTAGGCAGGGGGGCTGGGCGACCCTCCGTGCTGTGGCAGCACGTAAGAAGGG<br>AGAGCGCGGCGGCTGACAAGCGCCCCACTGTCAGACCCCGGAGTGATGTGACCCAC<br>AAGCAGCTCTCAGCATTTGGAGAGTATGTGGCTGAAATCTTACCCAAGTATGTCCAACA<br>AGTTCAGGTGTCCTGCCTTGATGAGTTAGAAATCTGTATCCATCCCGATGGAGTCATCC<br>CAACGCTGACTTTTCTCAGGGATCACACCAATGCACAATTCAAATCCTTGGCTGACTTG<br>ACGGCAGTGGATGTCCCAACTCGGCAGAACCGTTTTGAGATTGTCTACAACCTGCTGT<br>CTCTGCGGTTCAACTCTAGGATTCGTGTGAAGACCTATGCAGATGAGCTGACACCCATT<br>GACTCTATAGTGTCTGTGCACATCGCGGCCAATTGGTATGAGAGGGAGGTCTGGGACA<br>TGTTTGGAGTTTTCTTTTTTAACCACCCTGATTTAAGAAGGATCCTGACAGATTATGGCT<br>TCGAGGGACATCCTTTCCGGAAAGACTTTCCCCTCACTGGCTATGTTGAGCTTCGTTA<br>CGACGATGAGGTAAAGCGGGTAGTGGCTGAACCAGTGGAGCTGGCACAAGAATTCCG<br>CAAGTTTGACCTGAACAGCCCCTGGGAGGCTTTCCCTGCCTATCGCCAGCCTCCTGA<br>GAGTCTCAAGCTCGAAGCTGGAGACAAGAAGCCTGAAACCAAGTAATCTAGA
**Ndufs3-minigene:**<br>GATATCATTGTCTACAACCTGCTGTCTCTGCGGTTCAACTCTAGGATTCGTGTGAAGAC<br>CTATGCAGATGAGCTGACACCCATTGACTCTATAGTGTCTGTGCACATCGCGGCCAATT<br>GGTATGAGAGGGAGGTGAGTTACAGGATGTAGCGAGCCTTTCTGGGCCAGAAGTACA<br>GCATTGTAACTAACTATGGGGTGTGTCACAGTACAACTCCTCTGCTGTTGGCCCGATG<br>GACAGGTTTTTCTGAGAGCCACTCTTTTCTAGGTCTGGGACATGTTTGGAGTTTTCTTT<br>TTTAACCACCCTGATTTAAGAAGGATCCTGACAGATTATGGCTTCGAGGGACATCCTTT<br>CCGGAAAGACTTTCCCCTCACTGGCTATGTTGAGGTAGAAACCTTAAAACTGGGGTAG<br>CAGTCATGGCGCCTGGGAGATCATGACATGATGGGACTATGATCTGTAGATTATGGTGG<br>TTTAAGAGCCTGAGATGCAGTCCTAGTTCCATCTGGTAATGGGGCAAGTTGTTTGAATT<br>AGCCAAGGGACAGTTTTCCTATCTATATCATGGAAACAATAAGCACTTAAATATATGGAAA<br>AGTAAAGGCCAGTATGGAATCCTATACACTAGCTTATTGTGTGACCAAATGGATAGTATG<br>TCTGGATGTAGAGTGACCTAGCCTGGGACGAGTACTAAACAGAAGCCAAGGGAAAGG<br>GATAGGGCCTGGAGGAATCTGCACCTAGGCTGGTGCAGAACGGGCTCCTCAGTCATC<br>AGCCCTAACTTTTGCTTCTGCAGCTTCGTTACGACGATGAGGTAAAGCGGGTAGTGGC<br>TGAACCAGTGGAGCTGGCACAAGAATTCCGCAAGTTTGACCTGAACAGCCCCTGGGA<br>GGCTTTCCCTGCCTATCGCCAGCCTCCTGAGAGTCTCAAGCTCGAAGCTGGAGACAA<br>GAAGCCTGAAACCAAGTAACTCGAG

Table S3. Buffers

**Buffers used in isolation of primary brown adipocytes**	
Isolation buffer	1mg/ml Collagenase Type I (A004194, Diamond)
Primary culture medium	DMEM-high glucose (11965092, Gibco) 20% FBS (Gibco)
**Buffers used in adipocyte differentiation**	
Induction medium	DMEM, 10% FBS, 1μg/mL insulin (Sigma, I3536), 1nmol/L T3 (Sigma, T2877), 0.125 mmol/L indomethacin (Sigma, I7378), 1μmol/L dexamethasone (Sigma, D4902), and 1μmol/L rosiglitazone (Santa Cruz, sc202795)
Maintenance medium	DMEM, 10% FBS, 1μg/mL insulin (Santa Cruz: sc-29062), and 1nmol/L T3 (Sigma, T-2877)
**Nuclei isolation buffer**	
Nuclear lysis solution (NST)	0.1% NP40, 10 mM Tris-HCl, 146 mM NaCl, 1 mM CaCl2, 21 mM MgCl2 and 1 U/μl RNase inhibitor
ST Wash buffer	10 mM Tris-HCl, 146 mM NaCl, 1 mM CaCl2, 21 mM MgCl2, 0.01% BSA (NEB B9000S), and 40 U/mL RNase inhibitor
